# Beyond memory capacity: A probabilistic, dual store model of visuospatial working memory

**DOI:** 10.1371/journal.pcbi.1014535

**Published:** 2026-07-20

**Authors:** Eduardo A. Aponte, Thanneer M. Perumal, Francesca Cormack, Christopher H. Chatham

**Affiliations:** 1 Roche Pharma Research and Early Development, Roche Innovation Center Basel, F. Hoffmann-La Roche Ltd., Basel, Switzerland; 2 Cambridge Cognition, Cambridge, United Kingdom; University of Amsterdam: Universiteit van Amsterdam, NETHERLANDS, KINGDOM OF THE

## Abstract

The last decades have seen a great improvement in our understanding of visuospatial working memory (VSWM). Despite this progress, less is known about how information is stored, retained, and removed from VSWM when novel information is presented sequentially. Here, we present a novel computational model of the dynamics of VSWM that extends and improves classical ideas. Our analysis relies on data from three clinical trials involving neurotypicals and people with autism performing a smartphone based sequential VSWM task. In addition, we applied the model to data from a large clinical trial in prodromal Alzheimer’s disease. We demonstrate that visual information in our sequential task is stored in independent pools of contrasting resources, with perfect and imperfect retrieval rates. Our findings illustrate how computational models combined with remote cognitive testing are mature enough for applications in large-scale clinical research.

## Author summary

How does our brain store and retrieve visual information when we receive it piece-by-piece, rather than all at once? Here, we demonstrate how a detailed computational model of visuospatial working memory (VSWM) offers a clear answer to this question in the context of a task widely used in clinical research. We analyzed data from three clinical studies involving neurotypical and autistic individuals, and patients with prodromal Alzheimer’s disease. Participants performed the task both in a remote setting, using a gamified version of the task on their cellphones, as well as in the more traditional setting of lab testing. Our modeling clearly demonstrates that in this sequential visuospatial memory task, participants make use of different resources with dissimilar properties. Specifically, memory is stored in two systems: a small, highly reliable (deterministic) store and a larger, less reliable (stochastic) store where retrieval can sometimes fail. Our research provides a novel framework for understanding sequential VSWM and demonstrates that combining computational models with remote digital testing is a powerful tool for monitoring cognitive health in large-scale clinical research.

## Introduction

The last two decades have seen immense progress in our understanding of visuospatial working memory (VSWM), driven by novel and more refined experimental paradigms and computational models. These developments have focused on addressing a deceptively simple question: Is VSWM a discrete or a continuous resource? [1–8]. Two families of paradigms have received most attention: change detection tasks [6] and delayed reproduction tasks [4]. These paradigms have in common that all memoranda are presented to participants simultaneously. Yet, despite the intensive focus on these two paradigms, the mechanism of storing and retrieving information sequentially in VSWM has not been thoroughly investigated. Indeed, only but few studies have considered this question with great attention [9–13], as the interest on the interplay between the different cognitive resources required to solve complex tasks has started to spark more interest.

From a translational perspective, when moving from the lab to the clinic, our limited understanding of how sequentially acquired information is processed in VSWM represents a critical gap, as clinical studies often rely on dynamic tasks, rather than single-shot paradigms. One such task, initially developed by [14], and incorporated in the widely used CANTAB cognitive battery, has been used extensively in clinical and observational studies across conditions, including autism spectrum disorder (ASD; reviewed in [15]), healthy aging [16], mild cognitive impairment [17], dementia [18,19], major depressive disorder [20], and other psychiatric and neurological conditions. Thus, the absence of a satisfactory model of how information is sequentially encoded, maintained and retrieved from VSWM constitutes a significant gap in our computational understanding of clinical data across neurological and psychiatric conditions.

**Fig 1:**
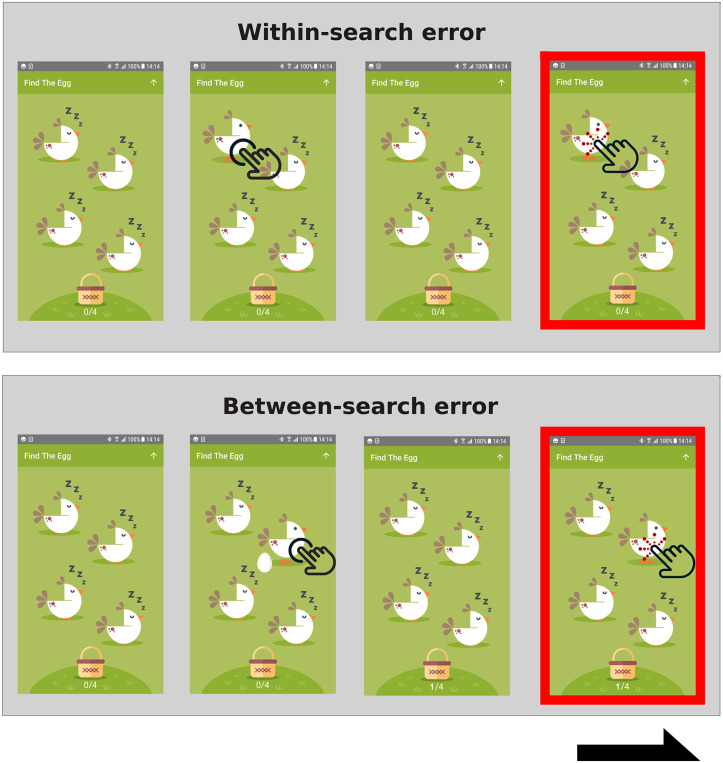
*Find the Egg* is a visuospatial working memory test for smartphones. Participants are instructed to tap on the chickens (items) to find the hidden eggs (targets). At any given time, only one chicken hides an egg. After an egg is found, the next one spawns behind a different chicken. Chickens hide exactly one egg per game. **Within-search errors** occur when participants tap twice an empty chicken before a new search has started. **Between-search errors** occur when participants tap on a chicken that previously hid an egg. Participants receive no feedback following an error.

With the goal of characterizing the mechanisms underlying this sequential memory task, here we developed a family of computational models of how information is progressively stored and retrieved from memory in Owen’s sequential task. First, we introduce several competing computational models of dynamic VSWM that make clear empirical predictions about participants’ performance. We then applied these models to data from *Find the Egg* (FtE) a gamified version of the task (Fig 1) deployed in two clinical studies involving participants of diverse ages, IQs and diagnoses (autism spectrum disorder and neurotypicals). Furthermore, we demonstrate that a specific index derived from our model can detect individual differences that robustly correlates with IQ, establishing both its convergent and discriminant validity. Finally, we apply the same computational approach to data from a large clinical trial in prodromal Alzheimer’s disease (AD) and demonstrate that the model can detect the subtle changes in cognition that occur early in AD pathology.

## Results

Here, we report data from FtE (Fig 1), a gamified digital assessment designed to evaluate cognitive performance through a sequential search task. This task is conceptually similar to the task originally proposed by [14]. In FtE, participants are instructed to search for targets (*eggs*) through a regularly spaced array of items (*chickens*) by tapping on them on a smartphone. Once a target is found, a *search* is completed, and a new search starts until all targets are found. Participants are informed that only one target is present at a time and that an item can only hide one target per game. Therefore, participants make an error when they tap items that already hid a target, or when they tap an empty item twice within the same search (see Fig 1). The former type of errors is called *between-search errors* (BSE) and the latter *within-search errors* (WSE). A round concludes successfully when a participant finds all targets. Once a round is completed, the next one starts with 2 additional targets on the screen, progressively increasing the difficulty of the task. A session ends either after the hardest condition (12 items) is completed or after a participant has made three consecutive errors. Because FtE was designed for weekly administration to individuals with a very wide range of cognitive abilities, it incorporates an adaptive design that modifies the difficulty (i.e., set size) according to participants’ performance. The set size for a new session is reduced by two items from the highest level achieved in the previous session. If a participant fails to complete the 4-item condition, the next session starts with the same 4 items.

In the rest of this section, we present increasingly complex computational models that explain and predict performance in this task. Specifically, we were interested in predicting the probability that a participant makes an error based on the number of items on the screen (set size) and the information that they have acquired during the course of the task. Then, we proceed to evaluate these models based on data collected from three different clinical trials.

### Discrete slot memory models

The main challenge that participants face in FtE is to avoid items in which a target was already encountered and items that have already been selected in an ongoing search. Optimal performance, therefore, depends on the ability to store, retrieve, and update information about already tapped items. To introduce our model and its notation, we build upon Cowan’s model of VSWM [5], which posits that VSWM consists of a finite set of slots in which novel discrete information is stored. Below we summarize this model in a number of assumptions, several of which we will revise later:

(1) Items are stored in a discrete number of slots, jointly referred to as the memory pool.
(2) An item occupies exactly one slot in memory. Memory capacity *M* is defined as the number of slots in the memory pool.
(3) New items are stored in memory as long as there are free slots in the memory pool.
(4) Items are stored and retrieved deterministically from memory.
(5) Participants only search items that are not in memory. We refer to these items as the decision pool.
(6) Participants select items from the decision pool randomly.

These assumptions enable an estimation of the probability of an error conditional on the memory capacity *M*, the set size *S* and the items previously searched. First, these assumptions imply that errors are only possible when the optimal number of items that need to be remembered *L* (for load) is larger than the memory capacity *M*. Second, when *L* is larger than *M*, the probability of an error is the probability of selecting a searched item from the decision pool. The size of the decision pool is simply the set size *S* minus the memory capacity *M*. Thus, we write


$$\begin{array}{c} P\left(Error|L\leq M,S\right)=\text{0}, \end{array}$$

$$\begin{array}{c} P\left(Error|L>M,S\right)=\frac{L-M}{S-M}, \end{array}$$

where *P(Error)* is the probability of an error.

Here, it is important to note that with this approach we seek to predict the probability that a participant will make an error, and which type of error, but not the probability that a specific item is selected. This limitation of the model is captured by the assumption that items are selected randomly from the decision pool, as the model does not predict which specific item will be tapped. Non-random strategies like, for example, first select items from the top of the screen, are fundamentally compatible with our approach, as they provide a further level of granularity that we don’t seek to model here.

The assumption that players only search items in the decision pool can be relaxed by postulating that there is a small but positive probability *η* of tapping any item on the screen. Thus, *η* accounts for errors caused by, e.g., attentional slips or motoric errors, which we collectively refer to as ‘lapses’. The implementation details follow the same approach as in [21,22] and are explained in S1 Text. For the sake of simplicity, we will omit this aspect of the model from the rest of the exposition.

To enable the derivation of the probability of BSE and WSE separately, we add the assumption that:

(7) All items are left out of memory with the same probability, i.e., all items have the same chance or priority to be stored in memory.

Accordingly, *on average*, the conditional probability of a BSE is equal to the number of items that hid an egg in previous searches (i.e., between-search items) *B* divided by the load *L*. Therefore, we can write


$$\begin{array}{c} P\left(BSE|M,B,S,L\right)=\left(\frac{B}{L}\right)\frac{L-M}{S-M}. \end{array}$$

Since the load *L* is simply the sum of *B* and *W*, it follows that


$$\begin{array}{c} P\left(WSE|M,B,S,L\right)=\left(\frac{L-B}{L}\right)\frac{L-M}{S-M}. \end{array}$$

**Fig 2:**
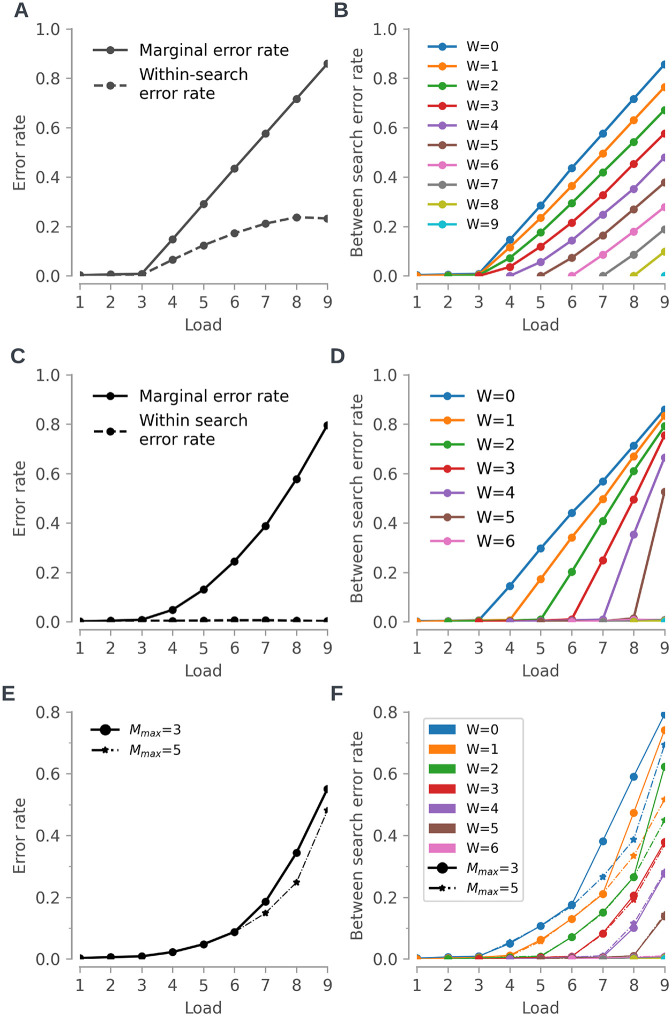
Model predictions in the set size S = 10 condition. Single store-model. **A)** Marginal and within-search error rate (WSE) rate as a function of load for *M=3*. The marginal error rate increases linearly with the load, i.e., the sum of the within- and between- search items (*W* and *B* respectively). **B)** Between-search error (BSW) rate as a function of load, for fixed values of W, depicted as lines in different colors. Dual-store model. **C)** Similar to A for a dual-store model (*M_B_* *=* *3, M_W_* *=* *10, η* *=* *0.03*). The marginal error rate is not a linear function of load. **D)** Similar to **B.** The dual-store model predicts that for a fixed *W*, the BSE rate increases linearly as a function of load. Stochastic multi-store model. Two simulations with *M_B_* *=* *3,* *M_W_* *=* *10, r* *=* *0.7, η* *=* *0.03*, and *M_stoch_* *=* *3* (solid lines and circles) and *M_stoch_* (dashed lines and stars). **E)** The marginal error rate is a non-linear function of load. The marginal error rate is only slightly different between *M_stoch_* *=* *3 and M_stoch_* *=* *5*. **F)** BSE rate similar to B and **D.** Contrary to a deterministic retrieval model (panel **D)**, BSE is a non-linear function of load for fixed *W***.**

Fig 2A-2B shows predictions from this model. Notably, the marginal error rate (the sum of BSE and WSE rates) is a linear function of the load *L*. In S2 Text and S1 Fig, we present two variations of this model in which between-search items are stored before all within-search items or vice versa. Importantly, in both cases, the marginal error rate is a linear function of load.

### Multiple-store models

Above we assumed that between- and within-search items occupy and compete for the same slots in a single store. However, the demands imposed by the task on each type of item are very different. Within-search items are retained only briefly and are regularly removed from memory once a new target is discovered. By contrast, between-search items are stored for longer periods and should not be overwritten across searches. This suggests that different cognitive processes may be involved in managing these two types of information within the task. To enable our model to capture these discrepant demands, we can relax assumption (1) to:

(1a) Between- and within-search items are stored in independent memory pools of capacity *M_B_* and *M_W_*.

Using the same logic as before, BSE are only possible when the number of between-search items *B* exceeds the between-search memory capacity *M_B_*:


$$\begin{array}{c} P\left(BSE|M_{B}\geq B,L,S\right)=\text{0}. \end{array}$$

In other case, the probability of a BSE is given by


$$\begin{array}{c} P\left({{BSE|M}_{B}<B,M}_{B},M_{W},B,W,S\right)=\frac{B-M_{B}}{S-M_{B}-min\left(M_{W},W\right)\;}. \end{array}$$

The denominator corresponds to the size of the decision pool, which is the set size *S* minus the total number of items in memory *M_B_* *+* *min* *(M_W_,* *W)*.

Importantly, the marginal probability of an error is no longer a linear function of the load *L* *=* *B* *+* *W* (Fig 2C), yet this dual-store model predicts that for a fixed number of within-search items *W*, the probability of a BSE is a linear function of the number of between-search items *B*, as displayed by Fig 2D.

### Stochastic memory retrieval

The models above share the assumption that participants can always correctly retrieve items stored in memory. However, on occasion, participants might not be able to *retrieve* an item from memory. Here, we can distinguish between *failures in retrieval* and *failures in encoding or storage*, in that the latter imply irreversible information loss, which *on average* would manifest as reduced memory capacity. By contrast, retrieval failures are reversible as information is not forgotten – rather, on every individual trial, there is a chance that information stored in memory is not correctly retrieved.

To enable the model to distinguish between retrieval and encoding/storage failures, we relax our assumption of deterministic retrieval to the following:

(4a) Participants can always correctly retrieve a limited number of items from memory *M_B_*. The remaining items are stored in *M_stoch_* slots, from which retrieval is stochastic and fails with a constant rate *1−r, r* ∈ *[0,1]*, independently of the set size or difficulty. Items not retrieved correctly from memory are part of the decision pool and therefore can lead to errors.

This assumption can also be understood as enabling non-discrete (i.e., partial) memory capacity. Specifically, the capacity of a slot can be formally understood as *r*, and its variability to be equal to *r(1−r)*, in analogy to non-discrete models [4].

Unlike the more restrictive models articulated earlier, this model predicts neither a linear marginal error rate as a function of load nor linear increases in BSE rate as a function of load for fixed *W* (Fig 2E-2F). Rather, it predicts three different phases *as a function of B*. Initially, errors can only be explained as *lapses* captured by parameter *η*, as *M_B_* items are perfectly stored and retrieved. Following this phase, items are kept in a store of maximum capacity *M_stoch_*, from which items are correctly retrieved at rate *r*. In the third phase, no more memory is available (*B* *>* *M_B_* *+* *M_stoch_*) and the error rate quickly increases as a function of *B* (Fig 2F). The formalization of this model is presented at length in the Methods section.

An important challenge in estimating the parameters of this model is that *r* and *M_stoch_* are linearly correlated since the expected total number of remembered items *M_total_*, takes the form


$$\begin{array}{c} {M_{total}=M}_{B}+rM_{stoch} \end{array}$$

when *B* *>* *M_B_* *+* *M_stoch_*, as *rM_stoch_* is the expected value of the underlying binomial process. To mitigate the effects of this collinearity, we focus the inference on the total memory capacity *M_total_*, rather than on its constituent elements.

### Measuring visuospatial working memory in clinical populations

The models of VSWM presented here are elaborations of classical models of working memory. Critically, we have shown how a hybridization of classical assumptions entails distinguishable predictions. Next, we evaluate these competing models of VSWM in clinical data, collected from different populations and age ranges. First, we evaluate the models presented above on the data from two clinical trials in ASD (oRBiting and V1aduct) described in detail in the methods section. Here, we briefly summarize both studies.

oRBiting (NCT03611075) was a non-drug study seeking to characterize clinical scales to measure repetitive and restrictive behaviors across ASD sub-populations. Participants were stratified in three age groups: children (5–12 years old), adolescents (13–17) and adults (18–45). oRBiting included both individuals with intellectual disability (IQ < 70) and IQ ≥ 70. Demographics breakup is reported in S2 Table. The study enrolled 144 participants, from which 22 were excluded because less than 4 sessions of FtE were available. FtE was administered every five days. The mean BSE and WSE rates were 19%(±8) and 2.5%(±2.7) respectively. After controlling for age, IQ was significantly correlated with BSE rate (*ρ* *=* −*0.30,* *P* *=* *0.001*) but not with the mean WSE rate (*ρ* *=* −*0.13,* *P* *=* *0.149*). Performance in the task is summarized in S2-S3 Figs and S2 Table.

V1aduct (NCT03504917) was a double blind, placebo controlled, 24-week phase III clinical trial investigating the safety and efficacy of *balovaptan*—an antagonist of the AVPR1A receptor*—*on adaptive behavior in autism [23,24]. After completing the 24 weeks, participants were offered to participate in an open-label study extending for up to 104 weeks. V1aduct was stopped after around half of the participants completed 24 weeks because of futility, as the study was deemed unlikely to reach its primary endpoint [24]. Data from the placebo and treatment arms were combined in all the following analyses, as there is no evidence of a treatment effect across several secondary analyses [24].

Here, we present data from a digital biomarker sub-study comprising 29 individuals who consented to use Roche’s ASD digital biomarker suite. Participants were provisioned with a smartphone configured to administer FtE and other cognitive tests every five days. At any moment, participants could opt out of this sub-study, which led to large differences in the number of FtE sessions available per participant (S2 Table). Three individuals were not included in the final analysis, as they participated in fewer than 4 sessions. The mean IQ of the rest of the participants (21 male) was 104 ± 16 (range 73–137) and mean age of 27 ± 16 years (range 18–62).

In V1aduct, we analyzed 33644 individual trials, ranging from as few as 131 trials per participant to as many as 3905. The longer duration of V1aduct and the factors mentioned above explain the large amount of data available for certain participants. IQ was correlated with mean BSE (*ρ* *=* −*0.50, P* *=* *0.008*) and mean WSE (*ρ* *=* −*0.42,* *P* = *0.030*) rates.

#### Modeling.

**Fig 3:**
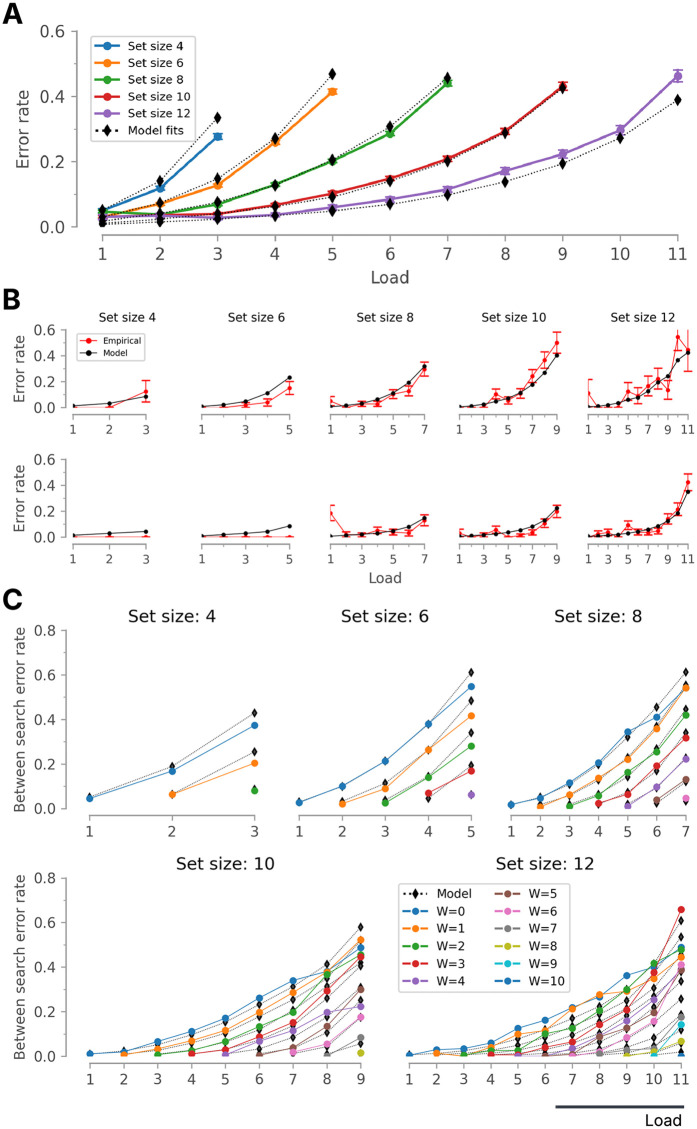
A) Mean marginal error rate from all participants in oRBiting. Error bars represent the standard error of the mean. The error rate is a nonlinear function of load. Dotted lines display the fits of the dual compartment stochastic memory retrieval model. B) Marginal error rate of two representative participants and model estimates, similar to A. Each row displays data from a representative participant. Clearly, the error rate is a nonlinear function of the load, ruling out the predictions of the single-store models. C) Between-search error rate as a function of load for fixed within-search item at different set sizes in oRBiting and model predictions averaged across all participants. W: Within-search items.

Were any of the models proposed above able to capture participants’ performance in FtE? To answer this question, we first examined the group level marginal error rate as a function of load across set sizes. Fig 3A demonstrates that, in contrast to the predictions of the single store models, the marginal error rate did not follow a linear trend. This was true at the level of single subjects (Fig 3B) and thereby this pattern is not an artifact of averaging across participants.

Given the very low number of WSE in both studies (less than 3% of all trials), in the following we assumed that participants’ memory capacity for within-search items was larger than the max set size, i.e., *M_W_>* 12. This implies that all WSE and a fraction of all BSE can be explained as lapses, quantified by parameter *η*.

**Fig 4:**
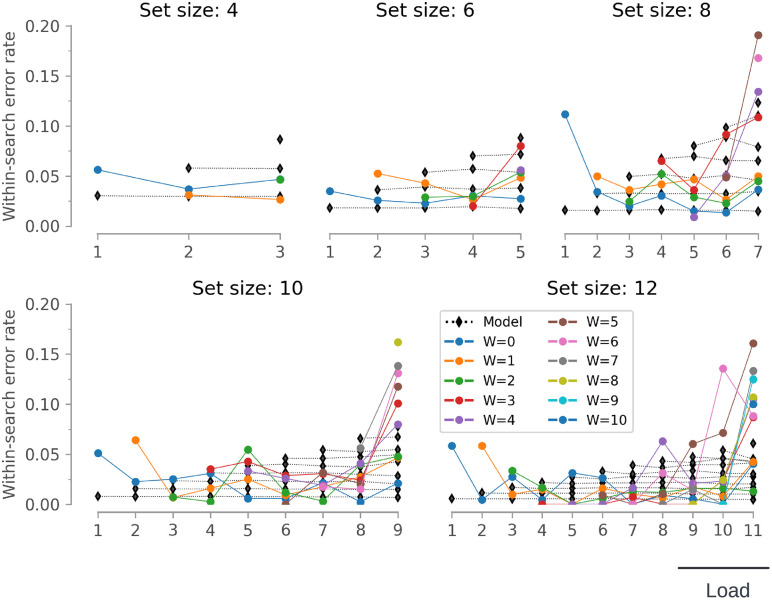
Within-search error (WSE) rate as a function of load for different set sizes (color lines) and model predictions (black, dotted lines). A key prediction of the multi-store model is that the WSE rate should be constant with increasing load, when *W* stays constant. Our analysis suggests that only in the most challenging conditions, the WSE rate increased with load for fixed ***W*.** WSE errors constituted only 3% of all the data. W=Within-search items.

The multi-store model makes a further prediction: the number of between-search items should not affect the WSE rate. The data overwhelmingly conforms to this prediction across loads (see Fig 4), with the exception of the highest loads, in which the WSE sharply increases with the number of between-search items for a fixed number of within-search items.

Qualitatively, we closely replicated our findings in V1aduct, as shown in S4 Fig.

S5 Fig displays a pooled analysis of both studies.

The previous analyses indicate that between- and within-search items are stored independently but leave open whether a distinction between *deterministic* and *stochastic* retrieval may better capture participants’ behavior in FtE. There are two main alternatives to the stochastic retrieval model proposed above: Items are always retrieved without error (*M_stoch_* *=* *0*); or no items are retrieved perfectly (*M_B_* *=* *0*).

Models were fitted quantitatively using the Hamiltonian Monte Carlo (HMC) algorithm as implemented in TensorFlow Probability [25]. HMC is a hybrid algorithm that uses the gradient of a target distribution to efficiently sample from it. The gradient computation is automatically handled by TensorFlow, using automatic differentiation. The target probability of the models is the trial-by-trial likelihood multiplied by the prior probability of the model parameters, as specified in the *Methods* section. The full model has two discrete parameters *M_B_* and *M_stoch_*, which could range from 0 to the largest set size possible in the task in addition to two continuous parameters: the probability of a slip *η* and the retrieval rate *r*. Since the HMC algorithm can only be used on continuous parameters, the discrete parameters can be easily marginalized out by computing


$$\begin{array}{c} p\left(y|\eta ,r\right)p(\eta ,r)=p(\eta ,r)\sum_{s=\text{0}}^{\text{1}\text{2}}\sum_{b=\text{0}}^{\text{1}\text{2}}p\left(y|\eta ,r,M_{stoch}=s,M_{B}=b\right)p\left(M_{stoch}=s,M_{B}=b\right). \end{array}$$

Once samples from the marginal posterior probability of parameters *η* and *r* were drawn using the HMC algorithm, these were used to estimate *M_total_* and to make posterior model predictions. Data from all sessions of a single participant were pooled together to obtain individual posterior estimates that were used to predict participant’s response on a trial-by-trial basis. Details of the inference are presented in the *Methods* and the code used for model fitting is openly available at https://osf.io/3ufg5.

To compare these two alternatives, we fixed the corresponding model parameter and compared the resulting Watanabe-Akaike Information Criterion (WAIC), a measure of model parsimony [26] that penalizes models for their complexity. In our two data sets, the full model explained participants’ data the best (Table 1). Removing the ability of the model to retrieve some of the items perfectly (by setting *M_B_* *=* *0*), or to retrieve some items imperfectly (by setting *M_stoch_* *=* *0*) deeply hurt the parsimony of the model. S6 Fig shows how the full model yielded qualitatively the best fits. We also calculated the relative probability of each model on the individual participant level. The mean relative probability of a model across participants represents the expected fraction of individuals in which that model is favored. Overall, the full model was favored by between 61 and 65% of participants, with the model without deterministic storage being favored by between 29 and 34% of participants. Thus, most participants used some form of non-deterministic retrieval, and the majority also relied on perfectly reliable storage.

**Table 1:** Quantitative model comparison.

Model	oRBiting		V1aduct	
	ΔWAIC	Mean model probability (SEM)	ΔWAIC	Mean model probability (SEM)
$M_{stoch}=\text{0}$	2080	0.04 (0.01)	1228	0.03 (0.07)
$M_{B}=\text{0}$	1189	0.29 (0.03)	234	0.34 (0.02)
Full model	0	0.65 (0.03)	0	0.61 (0.08)

Difference in Watanabe-Akaike Information Criterion (ΔWAIC) and posterior relative model probability for three models. Lower WAIC values indicate more parsimonious models. The mean model probability is the mean relative model probability across all participants. SEM: Standard error of the mean.

To investigate if there were differences between the models across groups in oRBiting, we applied MANOVA on the participants’ model probabilities transformed to the real interval. Participants with IQ below 70 were excluded, as this group was composed exclusively of autistic people. We found no effect of diagnostic group (P = 0.15), or age (P = 0.92).

In summary, our qualitative analysis showed that sequential recalling of items in FtE is mediated by two types of memory: one for within-search items and one for between-search items. These two types of memory are largely independent and only in the most challenging conditions are there signs of interference between them. Moreover, the recall of between-search items is characterized by both deterministic and stochastic retrieval.

### Individual differences

**Fig 5:**
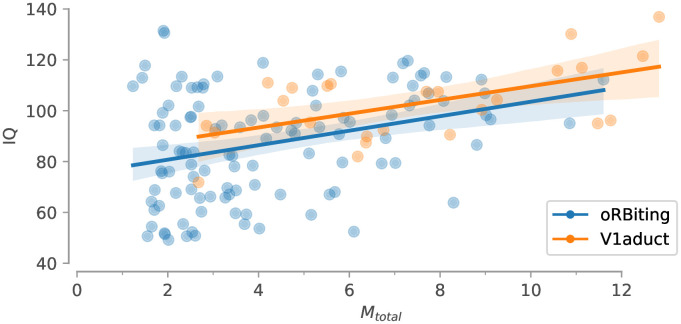
Total memory capacity $M_{total}$ correlated with IQ in both oRBiting (blue) and V1aduct (yellow).

Can total memory capacity *M_total_* shed light on participants’ overall cognitive skill? Since working memory is closely related to fluid intelligence [27–29], we hypothesized that total memory capacity *M_total_* should be associated with IQ across groups, independently of age. Indeed, in both studies we found a significant and similar correlation between IQ and *M_total_* (Fig 5, oRBiting: *r* *=* *0.32, η^2^_partia__l_* *=* *0.104, P* *<* *0.001* V1aduct: *r = 0.58, η^2^_partial_* *= 0.327, P = 0.003*).

### Alzheimer’s disease progression is associated with loss of memory capacity

Above, we demonstrated that *M_total_*, our model-based index of VSWM, is well associated with differences in IQ across age and neurodevelopmental diagnostic groups. More important from a clinical perspective is whether the model is sensitive to disease progression or treatment. To test this hypothesis, we analyzed longitudinal data from Scarlet RoAD (NCT01224106), a large, 104 weeks, Phase 3 clinical trial that assessed the safety and efficacy of gantenerumab in slowing the progression of AD in early stages of the disease [30]. The most relevant inclusion criteria were age between 50 and 85 years, classification of prodromal AD and presence of amyloid pathology (CSF *Aβ_1-42_* *<* *600* *ng/L*). Among others, participants were excluded in case of history of abnormal brain MRI and history of a neurological disease other than AD. Scarlet RoAD was terminated early for futility. At the time of termination, there was no significant difference in the primary endpoint between the placebo group and any of the active treatment groups. Hence, data from participants on active treatment and placebo were combined in the present analysis. Details about Scarlet RoAD have been published elsewhere [30].

Since this study started much before FtE was developed, we instead fitted the model to data from the CANTAB Spatial Working Memory task (CSWM) collected during the trial. While these two tests are structurally similar, there are several differences between them. Saliently, CSWM was administered during site visits and not remotely. Moreover, CSWM follows a fixed schedule of assessment, comprising of two rounds of 4, 6 and 8 items. Other differences between tasks are explained in the *Methods* section.

CSWM was administered at screening, baseline and weeks 24, 52, 76 and 104, in combination with several neuropsychological tests, including the ADAS-COG 13, the Mini Mental State Examination (MMSE)–two instruments designed to measure cognitive impairments–, and the Clinical Dementia Rating Scale Sum of Boxes (CDR-SB), a clinical endpoint used to measure both loss of function and cognitive impairment in dementia. In addition, volumetric T1 weighted images were collected (see *Methods*), from which hippocampal volumes, expressed as percentage of intracranial volume, were extracted. We used these clinical instruments, as well as changes in hippocampal volume, to assess the ability of *M_total_* to quantify changes in cognition in prodromal AD.

Given the longitudinal nature of our question, we extended the model to capture changes in working memory using a hierarchical approach, explained in detail in the *Methods* section. This hierarchical approach is essentially a random effects model that captures the correlations between sessions from a single individual while allowing for changes across sessions. Since the data from any individual session is limited, this extension of the model improves our ability to capture longitudinal changes in memory capacity. S3 Text and S8 and S9 Figs demonstrate the model’s accurate estimation of *M_total_* with parameter recovery tests. We fitted the model to trial-by-trial data from participants with at least five visits. Group and individual level fits are displayed in S10 and S11 Figs respectively, showing qualitative agreement in participants’ behavior across tasks and accurate model fits. In total, 371 participants were included in the final analysis (mean age 70 ± 7; 261 females). Additionally, we fitted this hierarchical model to data from oRBiting and V1aduct, obtaining estimates of *M_total_* for each session. No significant longitudinal change was detected (S12 Fig).

**Fig 6:**
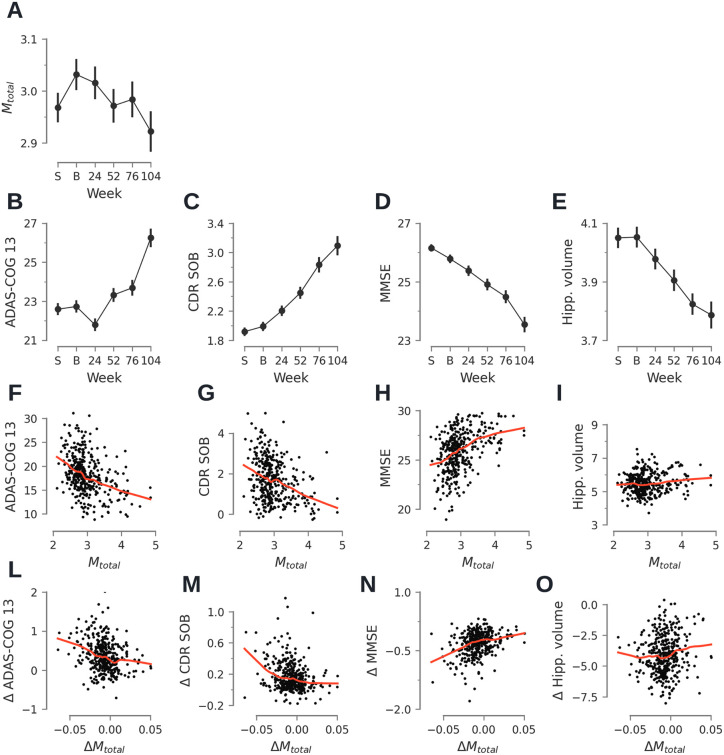
A-D) Mean value of *M_total_*, ADAS-COG-13, CDR SOB, MMSE and fractional hippocampal volume over Screening, Baseline, weeks 24, 52, 76 and 104. F-I) Estimated individual mean *M_total_* over all sessions except screening and mean individual values of clinical scales and hippocampal volume. Solid red lines depict moving average fit. L-O) Estimated individual slopes of change of *M_total_*, clinical scales and hippocampal volume. Slope of change of the hippocampal volume was scaled by a factor of 100 for clarity. Solid read lines depict moving average fits. ADAS-COG 13: Alzheimer’s Disease Assessment – Cognitive Subscale 13 Questions, CDR-SOB: Clinical Dementia Rating Scale, Sum of Boxes. MMSE: Mini Mental State Examination. S: Screening. B: Baseline. *M_total_*: Total memory capacity.

Once the model was estimated from the longitudinal data of every participant, an estimate of *M_total_* was extracted for each session. To test the longitudinal changes in *M_total_* and compare it with other clinical scales and hippocampal volume, we fitted mixed effect models with random intercepts and slopes for each participant, setting age and sex as covariates (see Fig 6A-6E). Exemplary individual longitudinal trajectories are displayed in S13 Fig. Following an uptick in *M_total_* after the screening session—likely due to familiarization with the test—there was a decline in *M_total_* over the next two years (Fig 6; *days in CT: t_345_* *=* −*0.25, P* *=* *0.011*). Since this effect was clearly attenuated by the familiarization with the test after the screening session, we repeated this test after excluding the screening session, which demonstrated a much larger effect size (*t_337_* *=* −*3.0, P* *< 0.001*). Quantitatively, the decline in working memory was small, but this was to be expected based on the early stage of the disease. The clinical scales and the hippocampal volume revealed a progressive decline in cognition and increased neurodegeneration in the same period.

Not only were the random intercepts of *M_total_* significantly correlated with other clinical endpoints (S3 Table; P < 10^-5^) but also their rate of change (Fig 6; P < 0.001). Interestingly, when we extended this analysis to the hippocampal volume (Fig 6H), intercepts were not significantly associated with *M_total_* (*ρ* *=* *0.06, P* *=* *0.21*), although there was a nominally significant but weak association between their slopes (*ρ* *=* *0.11, P* *=* *0.02*). By contrast, hippocampal volume was associated with the rest of the clinical scales, both in terms of participants’ intercepts and slopes (S4 Table). This suggests that while hippocampal integrity is associated with functional and cognitive changes in AD, these changes are independent of the loss of VSWM in early stages of the disease.

In summary, our model of VSWM can be extended to structurally similar tasks. Furthermore, it can characterize nontrivial aspects of working memory performance in both neurodevelopmental and aging populations. Moreover, it is sensitive to small longitudinal changes in VSWM—in this case, to changes caused by neurodegeneration —, which are well correlated with cognitive changes measured by the MMSE and ADAS-COG13. Nevertheless, loss of VSWM does not seem to be associated with reduction of hippocampal volume, suggesting a more secondary role for hippocampal function in the VSWM needed for this task.

## Discussion

Here, we have developed a family of models of VSWM that captures complex aspects of behavior in FtE and Owen’s paradigm—two closely related cognitive tasks—from a variety of participants. Our data originates from very diverse populations, including autistic children and adults with and without an intellectual disability, neurotypicals and adults with prodromal AD. Moreover, while some of the data was captured at home on smartphones, other data was collected in a controlled environment as part of a battery of cognitive assessments. The success of our model in explaining participants’ responses across indications and experimental conditions demonstrates the robust generalizability of our analytical approach. In addition, the main feature extracted from our model—total memory capacity—is associated both with fluid intelligence and with progressive cognitive deterioration in prodromal AD.

Our models represent a hybrid of classical and recent innovations in the understanding of working memory. We progressively elaborated our model starting from the initial assumption that visual information is stored in discrete and finite slots. In subsequent relaxations of this assumption, we allowed the retrieval of some items to be stochastic—effectively allowing for ‘slots’ with non-discrete capacity. This is in tension with the discrete-slot model’s assumption that after a certain fixed limit, ‘people must have a zero-information state about some of the items’ [31]. Thus, our model incorporates elements of discrete slot [1] and continuous models [8,32,33], and substantiates this hybridization through model comparison.

The notion that working memory relies on different stores dates to at least [34], who postulated that working memory emerges from the cooperation between primary and secondary memory. More recently, this distinction has been embraced by the individual differences literature [29,35–39]. According to this tradition, primary memory is a highly reliable and accessible store with limited capacity, while secondary memory is fundamentally limited by the ability to retrieve relevant information from it, not by its capacity. Whether VSWM relies on qualitatively different forms of memory has received scant attention, with most models postulating a single store model whose properties have been intensely debated. An exception is [37], who, based on individual differences, concluded that VSWM as measured by change detection paradigms is mainly related to secondary memory. However, [37] proposed no formal model of how primary and secondary visuospatial memory interact.

Our work adds to this tradition by formalizing and demonstrating separate stores of differential reliability and capacity for items in Owen’s sequential working memory task. In other words, sequential visuospatial information is retained in several ‘compartments’. Moreover, in the case of between-search items, some items are perfectly stored and retrieved, while others are retrieved at a limited rate, as demonstrated by quantitative model comparison. This closely matches the defining characteristics of primary and secondary memory.

Interestingly, we found that at very high loads, the number of WSE sharply increases with the number of between-search items. This constitutes a true violation of the assumption of two independent stores. This could indicate that there is no complete differentiation between the two memory stores or that there is some flexibility on how resources are allocated depending on the difficulty of the task. Unfortunately, these errors constitute a very small fraction of the data precluding their rigorous analysis.

There are several key advantages of the modeling approach presented here over raw descriptive statistics such as BSE and WSE rates. The latter are particularly problematic when reported across various set sizes as a low between-search error rate in the easiest condition is less telling than the same error rate in the hardest condition. By contrast, our model offers a simple, interpretable metric (*M_total_*) which integrates data across set sizes and can be extracted independently of the design of the test.

From a clinical research perspective, few studies have selectively examined deficits in VSWM in AD populations. Starting with early studies in familial AD, several experiments in carriers of PSEN2 and APP mutations suggested that preclinical and symptomatic AD is associated with reduced VSWM and that this deficit is due to reduced feature binding [40, 41] but see [42]. This deficit correlates with hippocampal integrity [40, 42], as reflected by a rare form of autoimmune encephalitis affecting the medial temporal lobe [43]. Here, we demonstrated longitudinal declines in VSWM in prodromal AD. Importantly, the rate of decline is associated with other cognitive and functional metrics, but not with the rate of hippocampal volume loss. This finding is intriguing, as it suggests that areas beyond the hippocampus play a key role in VSWM [44]. Thus, the decrease in VSWM detected by our model is likely to be associated with neurodegeneration outside the hippocampus.

Most participants in the three studies considered here were clinically diagnosed with autism or MCI/early AD, with only a small minority being considered neurotypicals. While the relative model probability did not differ significantly between ASD and neurotypicals, futures studies are needed to confirm and extend this initial observation.

There are important limitations to the approach we followed. FtE and similar paradigms were not designed to fully disentangle the complex interplay of different cognitive abilities that jointly constitute VSWM. In this regard, FtE stands in direct contrast to the paradigms traditionally used in non-clinical research, which tap on very circumscribed abilities with the aim of dissecting their underlying components. Such paradigms prioritize experimental control, simplicity and interpretability. By contrast, FtE is administered at home via a mobile phone with the intent of bringing computational models of working memory into greater contact with applied clinical research in the context of clinical trials.

Several aspects of how participants solve Owen’s task are not captured in our framework. For example, already [14] recognized that participants tend to start searches from the same item and follow stereotypical patterns while tapping items that they believed are empty. This strategy is considered to reflect ‘executive control’, rather than memory capacity [45].

A further factor not integrated in the current version of our model is participants' limited maintenance of information over the course of the task. This factor is likely to account for the consistent underestimation of participant’s error rate in trials that require particularly high maintenance (S7 Fig). Other aspects of the task that are also not covered by the model are recency effects on between-search items, whereby the last correctly tapped item is less likely to lead to an error.

In general, the models proposed here do not predict which specific items are tapped, but what is the probability that the next tap of a participant will be an error. Thus, our model cannot capture phenomena like ‘strategic’ searching, and the recency effect. While this feature of the model seems to have a limited effect on the ability of the model to predict errors in the task, this could explain small but systematic biases in the predictions of the model, as observed in Figs 3A and S8.

Above, we mentioned that total memory capacity *M_total_* is a metric that can be used to summarize participants’ performance in a single quantity. While useful from a translational perspective, this quantity cannot unveil the sources of errors on an individual or group level. Indeed, the structure of the model combined with the logic of the task makes it almost impossible to distinguish between errors caused by, for example, poor retrieval compared to low *M_stoch_*. Thus, the deep characterization of the deficits observed in AD is not possible here, for which more tailored tasks are needed. Nevertheless, *M_total_* was associated with both cognitive and functional endpoints (ADAS-Cog 13, CDR-SOB) in AD, indicating that, despite possible limitations in its psychometric properties, it reflects constructs that could matter to patients and their caregivers. Further work will be needed to explore these associations in AD and other indications.

Beyond its application as a digital/computational biomarker of disease progression in AD, it might be possible to use this approach to screen for the subtle changes in cognition that characterize mild cognitive impairment. This type of biomarkers could serve as a scalable and inexpensive approach to gate more in-depth or invasive diagnostic procedures, such as clinical cognitive testing or amyloid PET [46, 47]. It remains an open question whether computational approaches that model the cognitive processes behind measurable behavior will indeed improve the sensitivity and specificity of digital biomarkers of cognition.

The present work showcases a key application of computational psychiatry: long term monitoring of disease progression. By fusing tools from digital health for remote and frequent monitoring with interpretable computational modeling, we demonstrate a method to sensitively monitor participants in clinical trials to obtain early signs of progression (or the lack thereof, as desired from disease-modifying drugs).

## Methods

### Ethics statement

Written informed consent was obtained from all subjects and/or their legal guardian(s) (in case of minors). A list of institutional review boards or ethics committee that approved each of the studies analyzed here is provided in S4 Text.

### V1aduct and oRBiting

Data from FtE were collected in two clinical trials: oRBiting (NCT0361107) and V1aduct (NCT03504917). Orbiting was a 12-week observational study seeking to characterize different scales to measure repetitive and restrictive behaviors in different ASD sub-populations over time. The most relevant inclusion criteria were age between 5 and 45 years, IQ above 50 as assessed by the Stanford-Binet Intelligence Scale Fifth Edition [48] and a parent or study companion that could accompany participants to the clinical visits. For autistic individuals, the ASD diagnosis was confirmed by the Autism Diagnostic Observation Schedule-2 (ADOS-2; [49]). Written informed consent or assent was obtained during screening. Participants were excluded among other reasons if a disease or condition could interfere with the study in opinion of the principal investigators, and for autistic participants, if a diagnosis of a syndromic form of ASD was present (e.g., fragile-X syndrome, Angelman Syndrome, Prader-Willi Syndrome).

V1aduct was an interventional, 24-week Phase 3 clinical trial evaluating the efficacy and safety of balovaptan, an antagonist of the AVPR1A receptor. Details about V1aduct have been published elsewhere [24]. All participants were adults with a confirmed ASD diagnosis by the ADOS-2 and IQ equal to 70 or above as assessed by the Wechsler Adult Intelligence Scale-II [50]. The most relevant exclusion criteria were the intake of any drug that could cofound balovaptan’s hypothesized effect, pregnancy or breast feeding, substance use disorder as per DSM-5, epilepsy, seizures or an unstable psychotic or neurological disorder. After completing the 24-week study, participants could enroll in a 104-week open label study. Written Informed consent was obtained from all participants during screening. V1aduct was stopped after 322 participants were enrolled because of futility [24]. A small group of participants (35) were invited to participate in a digital biomarker sub-study including the FtE that extended into the open label study.

At the start of both trials, a study coordinator handed out a smartphone to participants or their study companion and instructed them on how to complete the tasks delivered through the phone. According to the schedule, FtE was administered every fifth day. At every point, participants had the option to deactivate FtE, which led to variable amounts of data collected from each participant.

### Experimental procedure: *Find the egg*

Fig 1 summarizes *Find the Egg*. Non-spatially overlapping items (chickens) appeared arranged on a regular grid on the screen of a smartphone in sets of 4, 6, 8, 10, or 12 items. Participants’ instructions were to find the hidden targets (eggs) by tapping on the items (chickens). Once a target was discovered, subjects had to drag it to the basket in the bottom of the screen. To complete a block, participants had to find all the hidden items. At no moment, participants received explicit feedback about their performance except for the total number of targets found so far.

FtE incorporates several changes with respect to [14] task, aimed at keeping the test challenging and interesting despite its dense administration schedule. First, when participants successfully completed a round, the difficulty of the task increased by displaying two extra items on the screen in the next round. Sessions ended after participants made more than three consecutive errors or after the most difficult condition (12 items) was completed. In the next session, the number of items on the screen was reduced by two compared to the highest accomplished level in the previous session. In case a participant failed to complete the easiest condition (4 items), the next session started with the same number of items.

### Scarlet RoAD

Scarlet RoAD was a multicenter, randomized, double-blind, placebo-controlled, Phase 3 clinical trial evaluating the safety and efficacy of gantenerumab, an anti-amyloid-beta monoclonal antibody, in slowing the progression of AD in prodromal patients. Details about this trial have been published before [30] and here we provide only a short summary of the most relevant aspects of Scarlet RoAD. Prodromal AD was asserted based on the criteria proposed by an International Working Group [51], and included an MMSE score ≥ 24, a CDR score of 0.5 together with memory box score of 0.5 or 1 and abnormal memory based on the Free and Cued Selective Reminding Test [52] and a Hachinski Ischemic Score ≤ 4 [53]. In addition, as evidence of amyloid pathology, cerebrospinal fluid levels of amyloid beta 1–42 below 600 ng/L were required. Scarlet RoAD was stopped because of futility after 797 individuals had been randomized.

Clinical scales (MMSE, CDR-SB and ADAS-COG 13) and the CANTAB were collected during screening, baseline and weeks 24, 52, 76 and 104. The CANTAB Spatial Working Memory Component (CSWMC) consisted of two training rounds of set size 4, followed by six games of set size 4, 6 and 8 items. The target (a yellow square) was randomly hidden behind one of the items (a different color square). As an additional rule, after a participant had searched two empty items, the location of the target was modified to be the next empty item searched. Thus, participants never had to remember more than two within-search items, reducing the length and complexity of the task. In contrast to FtE, there was no limit on the number of WSE and BSE so that a game only stopped after all targets were found. No further specific constraint applied to between-search items.

Hippocampal volume as a percentage of total intracranial volume was computed from 3D T1-weighted gradient images acquired mostly from 1.5 Tesla MRI scanners, and in exceptional cases, from 3.0 Tesla scanners. Segmentation was performed with the MIPAV software package [54].

The average clinical scales, hippocampal volume, and total memory capacity and their rate of change were estimated using mixed effect models with age and sex as fixed effects. Each participant’s behavior was modeled through a random intercept and a random slope modeling the change over the course of the trial. Correlations between outcomes were calculated from participants estimated random intercepts and slopes. S8 Fig displays representative data from six participants.

### Stochastic retrieval

Formally, we divide between-search memory into a deterministic store with capacity *M_B_* and a stochastic store from which *M_S_* items can be retrieved. Again, the conditional probability of a BSE when *B* *≤* *M_B_* *+* *M_S_* is zero. When *B* *>* *M_B_* *+* *M_S_*, we have that


$$\begin{array}{c} P\left(BSE|M_{B},M_{S},M_{W},B,W,S\right)=\frac{B-M_{B}-M_{S}}{S-M_{B}-M_{S}-min\left(M_{W},W\right)\;}. \end{array}$$

The probability of *M_S_* correct retrievals depends on the failure rate 1−*r* and the number of slots in stochastic memory *M_stoch_*. Assuming that retrievals are independent events, it follows that


$$\begin{array}{c} P\left(M_{S}|M_{stoch},r\right)=(\begin{array}{c} M_{stoch}\\ M_{S} \end{array})r^{M_{stoch}-M_{S}}(\text{1}-r{)}^{M_{S}}. \end{array}$$

Moreover, we assume that unused capacity cannot be reallocated to increase the precision of the memory slots already in use, as noted before. Thus, the marginal probability of a BSE is


$$\begin{array}{c} \sum_{M_{S}=\text{0}}^{M_{used}}\frac{B-M_{B}-M_{S}}{S-M_{B}-M_{S}-\left(M_{w},W\right)\;}(\begin{array}{c} M_{used}\\ M_{S} \end{array})r^{M_{used}-M_{S}}(\text{1}-r{)}^{M_{S}}, \end{array}$$

$$\begin{array}{c} M_{used}=\text{min}\left(B-M_{B},M_{stoch}\right). \end{array}$$

While somewhat complicated, this formula corresponds to the marginal probability of a BSE for different memory capacities *M_B_+M_S_*, where *M_S_* items succeed at retrieval at rate *r*.

### Model estimation

The most complex model we introduced here comprises 5 parameters: *η, r, M_B_, M_stoch_* and *M_W_* (see Table 1). Although *M_W_*, the memory capacity for within-search items, is in principle an important parameter, WSE were very uncommon at around 3% of all trials and its estimation was not feasible. Thus, we fixed its value to be larger than the maximum set size possible (in FtE, 12 items). This implies that all WSE were explained as lapses by the *η* parameter.

For all other parameters, we used Bayesian model inversion to compute their posterior probability. As *M_B_* and *M_stoch_* are discrete parameters, their prior was a categorical distribution, whereas the logit transform of *η* and *r*  were assumed to be Gaussian.

The Markov Chain Monte Carlo method was used to sample from the posterior distribution of the model parameters. Since the model has only two discrete parameters (*M_B_* and *M_stoch_*) with a small range, we sampled from the unnormalized marginal posterior distribution


$$\begin{array}{c} p\left(y|\eta ,r\right)p(\eta ,r)=\sum_{M_{B},M_{stoch}}p\left(y|{\eta ,r,M}_{B},M_{stoch}\right)p\left(M_{B},M_{stoch}\right)p(\eta ,r). \end{array}$$

In other words, we marginalized discrete parameters *M_B_* and *M_stoch_* and sampled exclusively the continuous parameters *η* and *r*. This allowed us to use the Hamiltonian Monte Carlo algorithm, a hybrid method that leverages the gradient of the posterior to propose samples. We ran four different chains and used the *R < 1.1* criterion to assess convergence. All routines were implemented using *tensor flow probability 0.24*.

The prior of the model consists of the distribution of the discrete parameters *M_B_* and *M_stoch_* and continuous parameters *η, r*∈ *[0,1]*. We defined a prior for individual η_S_ and r_S_, such that


$$\begin{array}{c} \sigma^{-\text{1}}\left(\eta_{s}\right)\sim N\left(H,\overset{\text{2}}{\underset{H}{\sigma }}\right),\;\sigma^{-\text{1}}\left(r_{s}\right)\sim N\left(R,\overset{\text{2}}{\underset{R}{\sigma }}\right), \end{array}$$

$$\begin{array}{c} \sigma (x)=\frac{\text{1}}{\text{1}+\;exp\;-x\;}. \end{array}$$

For *M_B_* and *M_stoch_*, we assumed that


$$\begin{array}{c} p\left(M_{B},M_{stoch}\right)=p\left(M_{B}\right)p\left(M_{stoch}\right), \end{array}$$

$$\begin{array}{c} p\left(M_{max}=i\right)=\frac{\text{1}}{\text{7}}\;for\;i<\text{7}\;and\;p\left(M_{max}=i\right)=\text{0}\;for\;i\geq \text{7}. \end{array}$$

The same prior distribution was used for *M_B_*. This model was used to fit data from oRBiting and V1aduct.

For model comparison, in addition to the WAIC, we computed the expected relative model probability of each model *M_i_*, for each participants’ data *Y* such that


$$\begin{array}{c} E\left[P\left(M_{i}|Y\right)\right]=E\left[Z^{-\text{1}}P\left(Y|M_{i}\right)P\left(M_{i}\right)\right] \end{array}$$

where the expected value is over all participants and Z^−^^1^ is a normalization constant such that


$$\begin{array}{c} \sum_{i}Z^{-\text{1}}P\left(Y|M_{i}\right)P\left(M_{i}\right)=\text{1}. \end{array}$$

Thus, for each participant, all three models were compared as per their model evidence *P(Y|M_i_)* *=* −*0.5WAIC* and normalized up to 1. We report the expected value of the normalized model evidence across all participants.

### Longitudinal models

The models presented before are well suited to represent the resources available to participants to store and retrieve visuospatial information but are not well suited to be sensitive to small longitudinal changes, typical in clinical applications. Thus, we made several modifications to the model above to capture the correlation between different sessions from a single participant, while allowing for different estimates of *M_total_* across sessions. This modified model was applied to data from Scarlet RoAD.

First, we simplified the model’s likelihood, by reducing it to a mixture model in which all possible memory capacities for between-search items *M_B_* are considered. The memory capacity for within-search items was assumed to be larger than the maximum set size in the game, and thus *M_W_>8*.

The probability of an action *y_l,t_* ∈ *{BSE,* *WSE,* *correct}*  in session *l*∈ *{1,...,L}* at trial t ∈ *{1,...,T_l_}* is given by the expression


$$\begin{array}{c} P\left(y_{t,l}|S_{l,t},B_{l,t},W_{l,t},\eta ,p^{l}\right)=\sum_{i=\text{0}}^{Z}P\left(y_{t,l}|M_{B}=i,M_{W}=\text{10},B_{l,t},W_{l,t},\eta \right)\overset{l}{\underset{i}{p}},\;\sum_{i=\text{0}}^{Z}\overset{l}{\underset{i}{p}}=\text{1}. \end{array}$$

Where Z represent the maximum set size in the experiment minus 1 and *p^l^* is the vector of probabilities of different memory capacities *M_B_*. Ignoring again the effect of any lapse, the probability of a between-search error if given by


$$\begin{array}{c} P\left({BSE|M}_{B}\geq B,W,S,\eta \right)=\text{0}. \end{array}$$

If *M_B_* *=* *i* *>* *B*


$$\begin{array}{c} P\left(BSE|M_{B}=i,B,W,S,\eta \right)=\frac{B-M_{B}}{S-M_{B}-W}. \end{array}$$

In other words, this is a mixture model with mixing probabilities *p^l^_i_* for each memory capacity*M_B_* *=* *i*. Note that we have assumed that all sessions share a single parameter *η*.

We modeled the correlations between sessions of the same participant by setting a hierarchical prior for the mixing probabilities *p^l^*. Specifically, we assumed that the probability of the mixture probabilities *p^l^* is given by the Dirichlet distribution


$$\begin{array}{c} p^{l}\sim Dirilecht(\alpha ),\;\alpha =\left\{\alpha_{\text{0}},\ldots ,\alpha_{Z-\text{1}}\right\},\;\alpha_{k}>\text{0}. \end{array}$$

Finally, the prior of *α* was set such that


$$\begin{array}{c} \alpha_{k}=\text{0}.\text{5}+L\rho_{i},\;\sum_{i=\text{1}}^{z}\rho_{i}=\text{1},\;\rho \sim Dirchlet(\omega ). \end{array}$$

Where *ω* is a vector of shape *Z*−*1* with all values fixed at 2 and *L* representing the number of sessions.

The central goal of this model is to estimate the posterior probability of *p^l^*. Assuming, posterior expected values


$$\begin{array}{c} \overset{l}{\underset{total}{M}}=\sum_{i=\text{0}}^{Z}i\overset{l}{\underset{i}{\hat{p}}}. \end{array}$$

This corresponds to the total memory capacity in session *l*.

This model allowed us to fit all sessions jointly, using *α* to represent the correlation between sessions of the same participant, while generating an estimate of *M_total_* on each session.

In S3 Text, we show that this model can be used to estimate *M*_*total*_ under a wide variety of conditions with high accuracy and minimal bias.

## Supporting information

S1 Text Lapses. (DOCX)

S2 Text Alternative priority models. (DOCX)

S3 Text Parameter recovery of hierarchical models. (DOCX)

S4 Text List of Institutional Review Boards & Independent Ethics Committees that approved oRBiting, V1aduct and Scarlet RoAD. (DOCX)

S1 Fig Simulated marginal error rate (ER) and within-search error (WSE) rate of priority models. Memory capacity was set to *M* *=* *4*. The marginal error rate increases linearly as a function of load in all models as all items compete for the same memory slots. The models differ in the predicted fraction of BSE and WSE.
(DOCX)

S2 Fig Behavioral Performance in oRBiting. **A)** Number of sessions completed. **B)** Maximum set size achieved. **C & D)** Associations between within- and between-search error rate and IQ (left and right panels).
(DOCX)

S3 Fig Compliance per week in V1aduct. (DOCX)

S4 Fig Error rate and model predictions in V1aduct. **A)** Marginal error rate and model predictions in V1aduct. B) Between-search error rates by load for fixed within-search items W for different set sizes in V1aduct.
(DOCX)

S5 Fig Error rate and model predictions in pooled oRBiting and V1aduct. **A)** Marginal error rate and model predictions. B) Between-search error rates by load for fixed within-search items W for different set sizes.
(DOCX)

S6 Fig Fits of three variants of the working memory model developed here to the combined data from oRBiting and V1aduct. In addition to the full model, we fitted models with the following constraints to the data from oRBiting and V1aduct: *M_stoch_* *=* *0 and M_B_* *=* *0*. Formal model analysis indicated that the full model had a large advantage compared to the more restricted models. Here, we display the fits of all the models to the oRBiting and V1aduct data sets combined. When *M_max_* *=* *0* and *M_B_* *=* *0*, the models overestimated the error rate. The full model produced more accurate fits. In each variant, one of the parameters was fixed to a specific value representing a particular set of assumptions. A) Stochastic memory slots *M_stoch_* were fixed to zero, that is, all memory was deterministic. B) Deterministic memory slots *M_B_* were fixed to zero. In this case, no items in memory were retrieved with perfect precision. C) Full model.
(DOCX)

S7 Fig Proportion of trials and root mean square error of model predictions as a function of trial in a game. A) Distribution of the trials as a function of set size and trial number in game. Trial number is proxy measure of memory maintenance, as it is an index of the duration of the game up to that specific trial. B) Root mean square error (RMSE) of the predicted error rate by the full model and the observed error rate as a function trial number. The RMSE clearly increases with trial number, indicating that memory maintenance plays a role in participants’ behavior that remains unexplained by the model.
(DOCX)

S8 Fig True vs. estimated memory capacity *M*_*total*_ across a wide arrangement of parameters. A) Estimates from 4 sessions. B) Estimates from 30 sessions.
(DOCX)

S9 Fig Parameter recovery as a function of sessions. A) Bias (mean difference between true total memory minus estimated value) as a function of the number of sessions. Bias was small but slightly increased with the number of sessions. B) Standard deviation of the error after correcting for the bias. C) One minus the slope of the regression of true vs estimated memory capacity (red lines in S4A-S4B Fig). Lower values indicate more accurate estimates.
(DOCX)

S10 Fig Group level fits from Scarlet RoAD. Individual trial-by-trial fits were averaged (black lines) after fitting trial-by-trial data. (DOCX)

S11 Fig Individual level fits from Scarlet RoAD from three representative participants. Each row presents data from an individual participant joint across all sessions available. Red lines represent mean and standard deviations of the error rate of three participants. Black dotted lines represent the model predictions after being fitted to individual trials.
(DOCX)

S12 Fig Longitudinal estimates of total memory capacity in oRBiting and V1aduct. The hierarchical model that accounts for possible longitudinal changes in total working memory was applied to data from oRBiting and V1aduct. The estimates from each session were entered into mixed effects model including age, IQ, gender and session as fixed effects and the baseline level and slope of change in total memory as random effects. There was no significant effect of session in total memory in any of these studies **A & B)** Estimated total memory capacity in oRBiting (A) and V1aduct (B) as a function of session. Faded lines correspond to individual estimates, solid black lines to the estimated marginal effect of session.
(DOCX)

S13 Fig Single subject trajectories of representative participants. Yellow circles represent single observations. Blue lines and shaded regions represent fit by a linear model fitted to individual observations and a 95% confidence interval respectively, while excluding the screening session.
(DOCX)

S1 Table Demographics oRBiting. (DOCX)

S2 Table Summary statistics behavioral outcomes oRBiting and V1aduct. Session duration refers to the total duration of a session in seconds. Sessions took on average less than 1.5 minutes to complete. Game duration refers to individual games, i.e., time required to find all the targets or until the game is stopped because of the number of consecutive errors.
(DOCX)

S3 Table Associations between *M_total_* and clinical scales and hippocampal volume. (DOCX)

S4 Table Associations between hippocampal volume and clinical scales. (DOCX)
