# Supplementary material for: Beyond memory capacity: A probabilistic, dual store model of visuospatial working memory
Source: PLoS Comput Biol. 2026 Jul 20;22(7):e1014535. doi: 10.1371/journal.pcbi.1014535 (PMC13475967; doi:10.1371/journal.pcbi.1014535)
Supplement: Supplementary file 1 [file pcbi.1014535.s001.docx]

Beyond memory capacity: A probabilistic, dual store model of visuospatial working memory

Supplementary materials

Eduardo A Aponte, Thanneer M Perumal, Francesca Cormack, Christopher H Chatham

## S1 Text: Lapses

We use the same mixture model approach as (Rouder et al., 2008, 2011) to explain errors that are due to lapses (e.g., attention slips, motoric errors, or similar stochastic sources of variance not adhering to the other processes we model). Thus, we assume that participants occasionally tap on a random item on the screen with probability $\eta$. When this occurs, the probability of a BSE or WSE is proportional to the number of between- or within-search items. Hence, assuming that $p_{m}$ is the probability mass function of an action defined by a model, the extended model takes the form

$$p\left( BSE \right)=\frac{\eta B}{S}+\left( 1-\eta\right)p_{m}\left( BSE \right),$$

$$p\left( WSE \right)=\frac{\eta W}{S}+\left( 1-\eta\right)p_{m}\left( WSE \right).$$

Note that $B/S$ and $W/S$ represent the probability of randomly tapping on a between- or within-search item, respectively. As we assumed that the memory capacity for within search items is unlimited, the probability of a WSE is

$$p\left( WSE \right)=\frac{\eta W}{S}.$$
