# Supplementary material for: Beyond memory capacity: A probabilistic, dual store model of visuospatial working memory
Source: PLoS Comput Biol. 2026 Jul 20;22(7):e1014535. doi: 10.1371/journal.pcbi.1014535 (PMC13475967; doi:10.1371/journal.pcbi.1014535)
Supplement: Supplementary file 2 [file pcbi.1014535.s002.docx]

Beyond memory capacity: A probabilistic, dual store model of visuospatial working memory

Supplementary materials

Eduardo A Aponte, Thanneer M Perumal, Francesca Cormack, Christopher H Chatham

## S2 Text: Alternative priority models

According to assumption 6, between- and within-search items are stored with the same priority in memory. There are two natural options to this premise:

(7a) Within-search items have the highest priority in memory.

(7b) Between-search items have the highest priority.

In the first case, between-search items are stored only after all within-search items are already in the memory pool. Thus, WSE are only possible when the number of within- search items *W* exceeds the memory capacity *M*:

$$P\left( WSE|M\geq W,L,S \right)=0,$$

$$P\left( WSE|M<W,L,S \right)=\frac{W-M}{S-M}.$$

We refer to this model as the *within-search priory* model. The *between-search priority model* can be derived using premise 6b.

S1 Fig shows predictions from these three models. Notably, the marginal error rate (the sum of BSE and WSE rates) is equal for all models and is a linear function of the load *L*. Nevertheless, these three models make different predictions regarding the fraction of WSE in each condition.
