# Supplementary material for: Beyond memory capacity: A probabilistic, dual store model of visuospatial working memory
Source: PLoS Comput Biol. 2026 Jul 20;22(7):e1014535. doi: 10.1371/journal.pcbi.1014535 (PMC13475967; doi:10.1371/journal.pcbi.1014535)
Supplement: Supplementary file 3 [file pcbi.1014535.s003.docx]

Beyond memory capacity: A probabilistic, dual store model of visuospatial working memory

Supplementary materials

Eduardo A Aponte, Thanneer M Perumal, Francesca Cormack, Christopher H Chatham

## S3 Text: Parameter recovery of hierarchical models

We evaluated the ability of the hierarchical model used to fit data from Scarlet RoAD to recover total memory capacity $M_{total}$ and the probability of a lapse $\eta$ by generating synthetic data from models with different sets of parameters as shown in Sup. Table 1. For these simulations, we assumed the same parameters across sessions and compared the mean $M_{total}$ across all sessions with the $M_{total}$ derived from the data generating parameters. In addition, we tested the effects of different numbers of sessions on the bias and accuracy of the parameter estimates.

**Parameters for synthetic data**

| **No. sessions** | $M_{B}$ | $M_{max}$ | $r$ | $\eta$ |
| --- | --- | --- | --- | --- |
| 4 | 0 | 0 | 0.1 | 0.03 |
| 8 | 1 | 1 | 0.3 |  |
| 15 | 2 | 2 | 0.5 |  |
| 30 | 3 | 3 | 0.7 |  |
|  | 4 | 4 | 0.9 |  |
|  | 5 | 5 |  |  |
|  | 6 | 6 |  |  |

List of parameters used for data generation. From each possible parameter combination, two different data sets were generated. In total, we analyzed 1960 artificial data sets.

S8 Fig displays the parameter estimates based on 4 and 30 sessions of FtE. Clearly, there is a small but consistent bias, defined as the mean difference between true and estimated memory capacity, in the parameter recovery. This bias increased with higher values of $M_{total}$, as demonstrated by a linear fit of the true and estimated values. Interestingly, the bias increased with the number of sessions fitted while the error of the estimates, after correcting for the bias, decreased with more data (S9 Fig). Overall, the error of the estimates was small, with a consistent and predictable bias.
