# Supplementary material for: Beyond memory capacity: A probabilistic, dual store model of visuospatial working memory
Source: PLoS Comput Biol. 2026 Jul 20;22(7):e1014535. doi: 10.1371/journal.pcbi.1014535 (PMC13475967; doi:10.1371/journal.pcbi.1014535)
Supplement: Supplementary file 4 [file pcbi.1014535.s004.docx]

Beyond memory capacity: A probabilistic, dual store model of visuospatial working memory

Supplementary materials

Eduardo A Aponte, Thanneer M Perumal, Francesca Cormack, Christopher H Chatham

# List of Institutional Review Boards & Independent Ethics Committees oRBiting

| **EC/IRB Name** | **EC/IRB Address** |
| --- | --- |
| Advarra IRB | 6940 Columbia Gateway Dr, Suite 110  Columbia, MD 21046-2878 |
| BRANY IRB | 225 Community Drive  Great Neck, NY 11021 |
| Advarra IRB | 6940 Columbia Gateway Dr, Suite 110  Columbia, MD 21046-2878 |
| Advarra IRB | 6940 Columbia Gateway Dr, Suite 110  Columbia, MD 21046-2878 |
| Advarra IRB | 6940 Columbia Gateway Dr, Suite 110  Columbia, MD 21046-2878 |
| Advarra IRB | 6940 Columbia Gateway Dr, Suite 110  Columbia, MD 21046-2878 |
| Nathan Kline Institute for Psychiatric  Research/Rockland  Psychiatric Center IRB | 140 Old Orangeburg Road,  Orangeburg, NY 10962 |
| Children's Hospital of  Philadelphia IRB | 3535 Market Street,  Philadelphia, PA 19104 |
| Holland Bloorview  Research Ethics Board | 150 Kilgour Road  Toronto Ontario M4g 1R8 |
| Wales Research Ethics  Committee 5 Bangor | Dinorwig Room, Neuadd Reichel Building, Bangor  University, Ffriddoedd Road, Bangor LL57 2TR |
| Advarra IRB | 6940 Columbia Gateway Dr, Suite 110  Columbia, MD 21046-2878 |
| Advarra IRB | 6940 Columbia Gateway Dr, Suite 110  Columbia, MD 21046-2878 |
| Advarra IRB | 6940 Columbia Gateway Dr, Suite 110  Columbia, MD 21046-2878 |
| Advarra IRB | 6940 Columbia Gateway Dr, Suite 110  Columbia, MD 21046-2878 |
| Advarra IRB | 6940 Columbia Gateway Dr, Suite 110  Columbia, MD 21046-2878 |
| Western Institutional  Review Board | 1019 39th Ave SE Suite 120,  Puyallup, Washington 98374-2118 |
| Advarra IRB | 6940 Columbia Gateway Dr, Suite 110  Columbia, MD 21046-2878 |

# List of Institutional Review Boards & Independent Ethics Committees V1aduct

**IRB/EC and address**

Advarra, 6940 Columbia Gateway Drive, COLUMBIA, MD, 21046, UNITED STATES

Advarra, 6940 Columbia Gateway Drive, COLUMBIA, MD, 21046, UNITED STATES

Yale University School of Medicine - Human Investigation

Committee, 47 College Street, Suite 204, P.O. Box 208010, New

Haven, CT, 06520, UNITED STATES

Advarra, 6940 Columbia Gateway Drive, COLUMBIA, MD, 21046, UNITED STATES

Advarra, 6940 Columbia Gateway Drive, COLUMBIA, MD, 21046, UNITED STATES

Chesapeake Research Review; IRB, 6940 COLUMBIA GATEWAY DRIVE, SUITE 110, Columbia, MD, 21046, UNITED STATES

Advarra, 6940 Columbia Gateway Drive, COLUMBIA, MD, 21046, UNITED STATES

NKI/RPC IRB, 140 Old Orangeburg Road, Bld 57 Rockland

Psychiatric Center Pharmacy, Orangeburg, NY, 10962, UNITED

STATES

Rush University Medical Center; Rush University Medical Center Institutional Review Board, Office of Reserch Affairs, 1653 W.

Congress Parkway, CHICAGO, IL, 60612, UNITED STATES

The University of Chicago IRB, 5841 S. Maryland Ave., CHICAGO, IL, 60637, UNITED STATES

Advarra Inc., 300-372 Hollandview Trail, L4G 0A5, AURORA, Ontario, CANADA

Advarra, 6940 Columbia Gateway Drive, COLUMBIA, MD, 21046, UNITED STATES

Advarra, 6940 Columbia Gateway Drive, COLUMBIA, MD, 21046, UNITED STATES

Advarra, 6940 Columbia Gateway Drive, COLUMBIA, MD, 21046, UNITED STATES

Advarra, 6940 Columbia Gateway Drive, COLUMBIA, MD, 21046, UNITED STATES

Advarra, 6940 Columbia Gateway Drive, COLUMBIA, MD, 21046, UNITED STATES

Lehigh Valley Health Network IRB, 1019 39th Avenue SE, Suite 120, Puyallup, WA, 98374-2115, UNITED STATES

Advarra, 6940 Columbia Gateway Drive, COLUMBIA, MD, 21046, UNITED STATES

Lehigh Valley Health Network IRB, 1019 39th Avenue SE, Suite 120, Puyallup, WA, 98374-2115, UNITED STATES

Advarra, 6940 Columbia Gateway Drive, COLUMBIA, MD, 21046, UNITED STATES

Advarra, 6940 Columbia Gateway Drive, COLUMBIA, MD, 21046, UNITED STATES

Advarra, 6940 Columbia Gateway Drive, COLUMBIA, MD, 21046, UNITED STATES

North East - Tyne & Wear South Research Ethics Committee,

Western IRB, 1019 39th Avenue SE, Suite 120, PUYALLUP, WA, 98374, UNITED STATES

Western University Health Science Research Ethics Board, Support Services Bldg, Rm 5150, London, ON, N6G 1G9, London, Ontario,

CANADA

Western University Health Science Research Ethics Board, Support Services Bldg, Rm 5150, London, ON, N6G 1G9, London, Ontario,

CANADA

COMITATO ETICO CATANIA 1, VIA S. SOFIA 78, 95123, CATANIA, Sicilia, ITALY

A.O. Città della salute e della scienza di Torino; Comitato Etico, Corso Bramante 88/90, 10126, Torino, Piemonte, ITALY

Comitato Etico Referente per l'Area di Pavia, VIALE GOLGI 19, 27100, PAVIA, Lombardia, ITALY

Advarra, 6940 Columbia Gateway Drive, COLUMBIA, MD, 21046, UNITED STATES

Advarra, 6940 Columbia Gateway Drive, COLUMBIA, MD, 21046, UNITED STATES

Advarra, 6940 Columbia Gateway Drive, COLUMBIA, MD, 21046, UNITED STATES

Advarra, 6940 Columbia Gateway Drive, COLUMBIA, MD, 21046, UNITED STATES

Advarra, 6940 Columbia Gateway Drive, COLUMBIA, MD, 21046, UNITED STATES

Advarra, 6940 Columbia Gateway Drive, COLUMBIA, MD, 21046, UNITED STATES

# List of Institutional Review Boards And Independent Ethics Committees Scarlet RoAD

Foundation Ethics Assessment
Biomedical Research (Bebo)
Noorderstaete 34, 9402 XB
Assen, The Netherlands

Southwest Finland Exp Ethical Committee
Regional Ethics Examination Board in Lund
Scientific Ethics Committees
Capital Region

UZ Leuven Gasthuisberg
Committee on Ethics
Herestraat, 49
3000 Leuven
Belgium

Bellberry Human Research Ethics Committee
129 Glen Osmond Road
Eastwood
South Australia, 5063

Austin Health HREC, Research Ethics Unit
Northern Sydney Coast HREC
Royal North Shore Hospital
Pacific Highway
St. Leonards
2065 New South Wales
Australia

Bioethics Committee For Clinical Research
Durango 216, Colonia Roma
06700, Mexico City
Mexico

Mario Education Committee
Research, Training And Bioethics Of The Hospital University Of Saltillo
Calzada Francisco I. Madero # 1291, Zona Centro
25000 Saltillo,
Mexico

Ethics Committee of Research
Faculty of Medicine
Autonomous University of Nuevo León S/N
64460, Monterrey
Mexico

Committee of Bioethics for Clinical Research
Puebla # 422 Office 4
06700, Col. Roma Sur
Mexico

Committee on The Protection Of Persons Ile De France VI
Hôpital Pitié-Salpêtrière, 47, Boulevard De L'hôpital
75013 Paris
France

Ethical Committee Research Hospital (IRCCS) San Raffaele
Via Olgettina 60
20132, Milan
Lombardy, Italy

Multimedica Hospital (IRCCS)
Sesto San Giovanni, Via Milanese, 300
20099, Sesto San Giovanni
Lombardy, Italy

Provincial Ethical Committee
Modena, Via Del Pozzo 71
Emilia-Romagna, Modena
Italy

Ethical Committee for St. John of God Clinical Research Center
Via Pilastroni, 4
25125 Brescia
Italy

Ethics Commission Berlin
State Office for Health and Social Affairs
Fehrbelliner Platz 1
10707 Berlin

Oxfordshire Research Ethics Committee B (South Central - Oxford B)
Bristol HRA Center (Main Office)
Level 3, Block B
Whitefriars
Lewins Mead
Bristol, Avon
BS1 2NT

Royal Adelaide Hospital Research Ethics Committee
North Terrace, Level 3, Hanson Institute
5000, Adelaide
South Australia

Ethical Committee of Clinical Research
Hospital Clinic of Barcelona
Villaroel, 170, Planta 4, Escalera 8
08036, Barcelona
Spain

Ethical Research Committee
Hospital Clinic de la Santa Creu In Sant Pau
08025, Barcelona,
Spain

Ethical Committee of Clinical Research,
Institut Municipal d’Assistència Sanitària
88 Edifici PRBB
08003, Barcelona
Spain

Ethical Committee of Clinical Research
Hospital De Cruces
5 Plant Pellet Administration
Plaza de Cruces S / N
48903 Barakaldo
Biscay, Spain

Independent Committee for Ethics
Uriburu 774, 1st Floor
C1027APP CABA
Buenos Aires, Argentina

Independent Committee on Ethics and Research
Galvan 4102,
C1431FWO, Buenos Aires
Argentina

IRB Services
372 Hollandview Trail
Suite 300, L4G 0A5
Ontario, Canada

Centre for Investigation, Division of Health Sciences, Monterrey Institute of Technology and Higher Education,
Morones, Prieto # 3000, Edificio Cites 3er.
East Wing Floor
64710 Monterrey
Mexico

Ethics in Research on Human Beings
Federal University of Sao Paulo
Rua Botucatu 572 – 1º Spirits
04023-062, Sao Paulo
Brazil

Scientific Committee on Research and Ethics on Health HCPA
Rua Ramiro Barcelos
2350 - 2nd Spirits
90035-003, Porto Alegre
Brazil

Research Ethics Committee for Human Beings,
Avenida General Carneiro
181,Central Building
80060-900, Curitiba
Brazil

Committee on Internal Medicine and Review of the Medical Society
Hospital Angeles Culiaca
Blvd. Alfonso G. Calderon Velarde No. 2193
Pte. Urban Development Tres Rios, 80020, Culiacan
Sinaloa, Mexico

The Independent Ethics Committee (CIE) for Clinical Pharmacology
Pte. J. E. Uriburu 774, 1st Floor, C1027aap,
Buenos Aires,
Argentina

Clinical Research Ethics Committee Hospital of Terrassa,
Torrebonica, S / N
08227, Terrassa
Barcelona, Spain

Istanbul University Clinical Faculty
Arastirmalar Etik Kurulu
Çapa, Istanbul 34093

The Independent Ethics Committee (CIE) for Clinical Pharmacology
Hospital Italiano de Buenos Aires, Gascon 450, 1181,
Capital Federal,
Argentina

Committee of Ethics in Biomedical Research (CEIB)
Montañeses 2325, C1428AQK
Ciudad Autónoma De Buenos Aires
Argentina

Research Ethics Committee
Hospital Mãe De Deus, Rua Costa,
150, Boy God
90110-270, Porto Alegre
Brazil

Central Northern Adelaide
Health Service Ethics Of Human Research Committee
28 Woodville Road, Woodville South
5011, Adelaide
South Australia
Australia

Western Institutional Review Board,
1019 39th Avenue Se, Ste 120,
Puyallup, WA 98374

Bydgoszcz Bioethics Committee
Chamber of Lakarska, Powstancow
Warsaw 11, 85-681, Bydgoszcz,
Poland

Clinical Research Ethics Committee
Hospital Clinic I Provincial Barcelona
ECRIN, Spain

Russian Medical Military Academy n.a. S.M.Kirov
Neurology Department
Ulitsa Academika Lebedeva, 6
194044, St. Petersburg
Russian Federation

Interregional Institution of Healthcare
Clinical and Diagnostic Center
Ethics Commission at Faculty Hospital St. Anne
Pekarska 53, 656 91
Brno, Czech Republic

Wide Area Ethics Committee
Largo Brambilla 3
50139, Florence
Tuscany, Italy

Ethical Committee for Parma,
Via Gramsci, 14,
43126, Parma
Emilia-Romagna, Italy

University Hospital of Ancona
Lancisi-G. Salesi Di Ancona, Via Conca 71
60100, Torrette - Ancona
Marche, Italy

University Of California San Diego
Human Research Protection Program
8950 Villa La Jolla Drive
Suite A-208
La Jolla, CA 92037
United States

Ethics committee of Saint-Petersburg State Institution Of Healthcare
Saint Petersburg State Institution of Healthcare City Geriatric
Medico-Social Center
Fontanka Embankment, Bld 148
190103, Saint Petersburg
Russian Federation

Oregon Health And Science University,
3181 SW Sam Jackson
Park Rd, Portland
OR, 97239

Commission for Ethics for Clinical Research
Lisbon Health Park
Av. Do Brasil, 53 - Pav. 17-A
1749-004, Lisbon

Independent Ethics Committee, Rusculleda Foundation
Avenida Colon 2057
X5003DCE, Cordoba
Argentina

Ethics Comission, Centre of Neurological Care Ltd.
Jiraskova 1389
516 01, Rychnov nad Kneznou
Czech Republic

Scientific Ethics Committees
Holbergsgade 6,
1057, Copenhagen
Denmark

Seoul St. Mary's Hospital; IRB
222, Banpo-Daero
Seocho-Gu, 06591
Seoul, Republic of Korea

Samsung Medical Center; IRB
81, Irwon-Ro, Gangnam-Gu
IRB Office, M3, Main Building
135-710, Seoul
Republic of Korea

Seoul National University
Bundang Hospital IRB
82, Gumi-Ro 173 Beon-Gil, Bundang-Gu
463-707, Seongnam-Si Gyeonggi-Do
Republic of Korea

Asan Medical Center Ethics Committee
388-1 Pungnapdong
Songpa-Gu, 138-736
Seoul, Republic of Korea

Konkuk University Medical Center IRB
120-1 Neungdong-Ro
Hwayang-Dong, Gwangjin-Gu
IRB Office, B2
143-729, Seoul
Republic of Korea

Bioethics Committee Research Unit in Chronic Degenerative Diseases
Colomos 2292, Providencia
CP 44620, Guadalajara Jalisco
Mexico

City Clinical Hospital # 2 n.a. V.I. Razumovsky
Chernyshevsky street, 141
410028, Saratov
Russian Federation
