# Supplementary material for: Beyond memory capacity: A probabilistic, dual store model of visuospatial working memory
Source: PLoS Comput Biol. 2026 Jul 20;22(7):e1014535. doi: 10.1371/journal.pcbi.1014535 (PMC13475967; doi:10.1371/journal.pcbi.1014535)
Supplement: Supplementary file 5 [file pcbi.1014535.s005.docx]

Beyond memory capacity: A probabilistic, dual store model of visuospatial working memory

Supplementary materials

Eduardo A Aponte, Thanneer M Perumal, Francesca Cormack, Christopher H Chatham

**S1 Fig:** **Simulated marginal error rate (ER) and within search error (WSE) rate of priority models**. Memory capacity was set to $M=4$. The marginal error rate increases linearly as a function of load in all models as all items compete for the same memory slots. The models differ in the predicted fraction of BSE and WSE rates. ER: Marginal error rate. BSE: Between search error. WSE: Within search error.
