# Supplementary material for: Beyond memory capacity: A probabilistic, dual store model of visuospatial working memory
Source: PLoS Comput Biol. 2026 Jul 20;22(7):e1014535. doi: 10.1371/journal.pcbi.1014535 (PMC13475967; doi:10.1371/journal.pcbi.1014535)
Supplement: Supplementary file 6 [file pcbi.1014535.s006.docx]

Beyond memory capacity: A probabilistic, dual store model of visuospatial working memory

Supplementary materials

Eduardo A Aponte, Thanneer M Perumal, Francesca Cormack, Christopher H Chatham

**S2 Fig: Behavioral Performance in oRBiting. A)** Number of sessions completed. **B)** Maximum set size achieved. **C & D)** Associations between within- and between-search error rate and IQ (left and right panels). IQ: Intelligence quotient.
