# Supplementary material for: Beyond memory capacity: A probabilistic, dual store model of visuospatial working memory
Source: PLoS Comput Biol. 2026 Jul 20;22(7):e1014535. doi: 10.1371/journal.pcbi.1014535 (PMC13475967; doi:10.1371/journal.pcbi.1014535)
Supplement: Supplementary file 8 [file pcbi.1014535.s008.docx]

Beyond memory capacity: A probabilistic, dual store model of visuospatial working memory

Supplementary materials

Eduardo A Aponte, Thanneer M Perumal, Francesca Cormack, Christopher H Chatham

**S4 Fig**: **Error rate and model predictions in V1aduct.** **A)** Marginal error rate and model predictions in V1aduct. B) Between-search error rates by load for fixed within-search items W for different set sizes in V1aduct. W: Number of within-search items.
