# Supplementary material for: Beyond memory capacity: A probabilistic, dual store model of visuospatial working memory
Source: PLoS Comput Biol. 2026 Jul 20;22(7):e1014535. doi: 10.1371/journal.pcbi.1014535 (PMC13475967; doi:10.1371/journal.pcbi.1014535)
Supplement: Supplementary file 10 [file pcbi.1014535.s010.docx]

Beyond memory capacity: A probabilistic, dual store model of visuospatial working memory

Supplementary materials

Eduardo A Aponte, Thanneer M Perumal, Francesca Cormack, Christopher H Chatham

**S6 Fig: Fits of three variants of the working memory model to the combined data from oRBiting and V1aduct.** In addition to the full model, we fitted models with the following constraints to the data from oRBiting and V1aduct: $M_{stoch}=0 \mathrm{and}$ $M_{B}=0$. Formal model analysis indicated that the full model had a large advantage compared to the more restricted models. Here, we display the fits of all the models to the oRBiting and V1aduct data sets combined. When $M_{max}=0$ and $M_{B}=0$, the models overestimated the error rate. The full model produced more accurate fits. In each variant, one of the parameters was fixed to a specific value representing a particular set of assumptions. A) Stochastic memory slots $M_{stoch}$ were fixed to zero, that is, all memory was deterministic. B) Deterministic memory slots $M_{B}$ were fixed to zero. In this case, no items in memory were retrieved with perfect precision. C) Full model.
