# Supplementary material for: Beyond memory capacity: A probabilistic, dual store model of visuospatial working memory
Source: PLoS Comput Biol. 2026 Jul 20;22(7):e1014535. doi: 10.1371/journal.pcbi.1014535 (PMC13475967; doi:10.1371/journal.pcbi.1014535)
Supplement: Supplementary file 11 [file pcbi.1014535.s011.docx]

Beyond memory capacity: A probabilistic, dual store model of visuospatial working memory

Supplementary materials

Eduardo A Aponte, Thanneer M Perumal, Francesca Cormack, Christopher H Chatham

**S7 Fig: Proportion of trials and root mean square error of model predictions as a function of trial in a game.** A) Distribution of the trials as a function of set size and trial number in game. Trial number is proxy measure of memory maintenance, as it is an index of the duration of the game up to that specific trial. B) Root mean square error (RMSE) of the predicted error rate by the full model and the observed error rate as a function trial number. The RMSE clearly increases with trial number, indicating that memory maintenance plays a role in participants’ behavior that remains unexplained by the model.
