# Supplementary material for: Beyond memory capacity: A probabilistic, dual store model of visuospatial working memory
Source: PLoS Comput Biol. 2026 Jul 20;22(7):e1014535. doi: 10.1371/journal.pcbi.1014535 (PMC13475967; doi:10.1371/journal.pcbi.1014535)
Supplement: Supplementary file 12 [file pcbi.1014535.s012.docx]

Beyond memory capacity: A probabilistic, dual store model of visuospatial working memory

Supplementary materials

Eduardo A Aponte, Thanneer M Perumal, Francesca Cormack, Christopher H Chatham

**S8 Fig**: **True vs. estimated memory capacity** $\boldsymbol{M}_{\boldsymbol{total}}$ **across a wide arrangement of parameters.** A) Estimates from 4 sessions. B) Estimates from 30 sessions.
