# Supplementary material for: Beyond memory capacity: A probabilistic, dual store model of visuospatial working memory
Source: PLoS Comput Biol. 2026 Jul 20;22(7):e1014535. doi: 10.1371/journal.pcbi.1014535 (PMC13475967; doi:10.1371/journal.pcbi.1014535)
Supplement: Supplementary file 13 [file pcbi.1014535.s013.docx]

Beyond memory capacity: A probabilistic, dual store model of visuospatial working memory

Supplementary materials

Eduardo A Aponte, Thanneer M Perumal, Francesca Cormack, Christopher H Chatham

**S9 Fig: Parameter recovery as a function of sessions**. A) Bias (mean difference between true total memory minus estimated value) as a function of the number of sessions. Bias was small but slightly increased with the number of sessions. B) Standard deviation of the error after correcting for the bias. C) One minus the slope of the regression of true vs estimated memory capacity (red lines in S8 Fig A-B). Lower values indicate more accurate estimates. SD: Standard deviation.
