# Supplementary material for: Beyond memory capacity: A probabilistic, dual store model of visuospatial working memory
Source: PLoS Comput Biol. 2026 Jul 20;22(7):e1014535. doi: 10.1371/journal.pcbi.1014535 (PMC13475967; doi:10.1371/journal.pcbi.1014535)
Supplement: Supplementary file 15 [file pcbi.1014535.s015.docx]

Beyond memory capacity: A probabilistic, dual store model of visuospatial working memory

Supplementary materials

Eduardo A Aponte, Thanneer M Perumal, Francesca Cormack, Christopher H Chatham

**S11 Fig: Individual level fits from Scarlet RoAD from three representative participants.** Each row presents data from an individual participant joint across all sessions available. Red lines represent mean and standard deviations of the error rate of three participants. Black dotted lines represent the model predictions after being fitted to individual trials.
