# Supplementary material for: Beyond memory capacity: A probabilistic, dual store model of visuospatial working memory
Source: PLoS Comput Biol. 2026 Jul 20;22(7):e1014535. doi: 10.1371/journal.pcbi.1014535 (PMC13475967; doi:10.1371/journal.pcbi.1014535)
Supplement: Supplementary file 16 [file pcbi.1014535.s016.docx]

Beyond memory capacity: A probabilistic, dual store model of visuospatial working memory

Supplementary materials

Eduardo A Aponte, Thanneer M Perumal, Francesca Cormack, Christopher H Chatham

**S12 Fig: Longitudinal estimates of total memory capacity in oRBiting and V1aduct.** The hierarchical model that accounts for possible longitudinal changes in total working memory was applied to data from oRBiting and V1aduct. The estimates from each session were entered into mixed effects model including age, IQ, gender and session as fixed effects and the baseline level and slope of change in total memory as random effects. There was no significant effect of session in total memory in any of these studies **A & B)** Estimated total memory capacity in oRBiting (A) and V1aduct (B) as a function of session. Faded lines correspond to individual estimates, solid black lines to the estimated marginal effect of session.
