# Supplementary material for: Beyond memory capacity: A probabilistic, dual store model of visuospatial working memory
Source: PLoS Comput Biol. 2026 Jul 20;22(7):e1014535. doi: 10.1371/journal.pcbi.1014535 (PMC13475967; doi:10.1371/journal.pcbi.1014535)
Supplement: Supplementary file 17 [file pcbi.1014535.s017.docx]

Beyond memory capacity: A probabilistic, dual store model of visuospatial working memory

Supplementary materials

Eduardo A Aponte, Thanneer M Perumal, Francesca Cormack, Christopher H Chatham

**S13 Fig**: **Single subject trajectories of representative participants.** Yellow circles represent single observations. Blue lines and shaded regions represent fit by a linear model fitted to individual observations and a 95% confidence interval respectively, while excluding the screening session. ADAS COG: Alzheimer’s Disease Assessment Scale-Cognitive Subscale 13 Questions. CDR: Clinical Dementia Rating Scale, Sum of Boxes. MMSE: Mini-Mental State Examination. Hipp. volume: Hippocampal volume.
