# Supplementary material for: Beyond memory capacity: A probabilistic, dual store model of visuospatial working memory
Source: PLoS Comput Biol. 2026 Jul 20;22(7):e1014535. doi: 10.1371/journal.pcbi.1014535 (PMC13475967; doi:10.1371/journal.pcbi.1014535)
Supplement: Supplementary file 18 [file pcbi.1014535.s018.docx]

Beyond memory capacity: A probabilistic, dual store model of visuospatial working memory

Supplementary materials

Eduardo A Aponte, Thanneer M Perumal, Francesca Cormack, Christopher H Chatham

**S1 Table: Demographics oRBiting**

|  | **N** | **N male** |
| --- | --- | --- |
| ASD – IQ ≤ 70 |  |  |
| Children | 9 | 8 |
| Adolescent | 10 | 10 |
| Adults | 11 | 8 |
| ASD – IQ > 70 |  |  |
| Children | 14 | 12 |
| Adolescent | 13 | 10 |
| Adults | 12 | 9 |
| NT – IQ >70 |  |  |
| Children | 13 | 4 |
| Adolescent | 16 | 9 |
| Adults | 14 | 1 |

ASD: Autism spectrum disorder. IQ: Intelligence quotient. NT: Neurotypical.
