# Supplementary material for: Beyond memory capacity: A probabilistic, dual store model of visuospatial working memory
Source: PLoS Comput Biol. 2026 Jul 20;22(7):e1014535. doi: 10.1371/journal.pcbi.1014535 (PMC13475967; doi:10.1371/journal.pcbi.1014535)
Supplement: Supplementary file 19 [file pcbi.1014535.s019.docx]

Beyond memory capacity: A probabilistic, dual store model of visuospatial working memory

Supplementary materials

Eduardo A Aponte, Thanneer M Perumal, Francesca Cormack, Christopher H Chatham

**S2 Table: Summary statistics behavioral outcomes oRBiting and V1aduct**

|  | **oRBiting** | | **V1aduct** | |
| --- | --- | --- | --- | --- |
|  | Mean | SD | Mean | SD |
| Number of sessions | 14.261 | 6.920 | 22.360 | 18.624 |
| Session duration (s) | 76.813 | 61.867 | 60.403 | 57.717 |
| Game duration (s) | 22.316 | 9.055 | 19.496 | 5.033 |
| Trial duration (s) | 1.176 | 0.415 | 0.986 | 0.194 |
| Max set size | 10.304 | 1.540 | 10.769 | 1.367 |
| Between search error rate [%] | 19.031 | 8.012 | 17.027 | 8.303 |
| Within search error rate [%] | 2.485 | 2.628 | 1.696 | 2.250 |

Session duration refers to the total duration of a session in seconds. Sessions took on average less than 1.5 minutes to complete. Game duration refers to individual games, i.e., time required to find all the targets or until the game is stopped because of the number of consecutive errors. SD: Standard deviation. *s*: seconds.
