# Supplementary material for: Beyond memory capacity: A probabilistic, dual store model of visuospatial working memory
Source: PLoS Comput Biol. 2026 Jul 20;22(7):e1014535. doi: 10.1371/journal.pcbi.1014535 (PMC13475967; doi:10.1371/journal.pcbi.1014535)
Supplement: Supplementary file 20 [file pcbi.1014535.s020.docx]

Beyond memory capacity: A probabilistic, dual store model of visuospatial working memory

Supplementary materials

Eduardo A Aponte, Thanneer M Perumal, Francesca Cormack, Christopher H Chatham

**S3 Table****: Associations between** $M_{total}$ **and clinical scales and hippocampal volume**

|  | **Intercept** | |  | **Rate of change (slope)** | |
| --- | --- | --- | --- | --- | --- |
|  | $\rho$ | P |  | $\rho$ | P |
| ADAS COG 13 | -0.38 | $<{10}^{-5}$ |  | -0.25 | $<{10}^{-5}$ |
| CDR-SB | -0.26 | $<{10}^{-5}$ |  | 0.19 | $<{10}^{-3}$ |
| MMSE | 0.40 | $<{10}^{-5}$ |  | 0.28 | $<{10}^{-5}$ |
| Hippocampal volume | 0.06 | 0.21 |  | 0.11 | 0.02 |

P values are not corrected for multiple comparisons. ADAS COG 13: Alzheimer’s Disease Assessment Scale-Cognitive Subscale 13 Questions. CDR-SB: Clinical Dementia Rating Scale, Sum of Boxes. MMSE: Mini-Mental State Examination.
