# Supplementary material for: Beyond memory capacity: A probabilistic, dual store model of visuospatial working memory
Source: PLoS Comput Biol. 2026 Jul 20;22(7):e1014535. doi: 10.1371/journal.pcbi.1014535 (PMC13475967; doi:10.1371/journal.pcbi.1014535)
Supplement: Supplementary file 22 [file pcbi.1014535.s023.docx]

Dear Dr. Mejias,

We thank you and the reviewers for the supportive and constructive comments. Below, you will find the response to all comments. We have organized the answers by reviewer and comment. The original comments and questions are included in italics and our answers in regular text. We also included text that is newly included in the manuscript in indented, regular text.

Yours Sincerely,

Eduardo Aponte

# Editor Comments

All three reviewers found the work interesting and potentially suitable for publication in PLOS Computational Biology. They also identified several points that should be addressed in the revision, including some issues in the presentation of of the results and refinement of some claims, the absence of details in the methods and results section, and a more comprehensive assessment of some of the results. Please keep in mind that the code to replicate the model results should also be made available as per the PLOS Data Policy guidelines.

Reviewer's Responses to Questions

For simplicity, all questions and comments are included in the current file in italics.

## Reviewer #1

### Comment 1

*I believe that the results presented here are interesting and useful. The authors have used a computational modelling approach to capture the pattern of errors seen in the “Find the Egg” task and CANTAB Spatial Working Memory task. Importantly, both of these tasks require participants to search between items while holding in memory information about targets during previous searches (between search items) and also information about items previously searched within the current search (within search items). A modelling approach that accounts for these two types of storage, as well as allowing for separate storage v retrieval errors, provided the best account across two datasets.*

We thank the Reviewer for the supportive words.

### Comment 2

*My main concern relates to how these results are presented. The abstract describes how the model explains how visual information is stored “in sequential tasks”. The Authors Summary asks “how does our brain store and retrieve visual information when we receive it piece-by-piece, rather than all at once?” The introduction describes how “our limited understanding of how sequentially acquired information is processed in VSWM represents a critical gap”. I believe this is misleading. The authors have successfully modelled one very specific type of sequential task; one that involves a search task and the need to retain both within and between search information. Arguably, most sequential VSWM tasks do not involve this requirement. Instead, participants are asked to remember independent sequences of items (eg. the Corsi block task), or monitor a stream of sequentially presented items (eg. a visuo-spatial n-back task). The multi-store modelling approach presented here can not be used for tasks such as these, because they do not involve remembering within as well as between search/session items.

Statements like this “We demonstrate that visual information in sequential tasks is stored in a variety of ways that impose contrasting constraints, showing the existence of pools of independent resources, with perfect and imperfect retrieval rates” (abstract) and “In summary, our qualitative analysis showed that sequential recalling of items is mediated 288 by two types of memory: one for within-search items and one for between-search items” (page 20, line 287) are particularly misleading, as the results only relate to a specific type of sequential task, not sequential tasks in general.*

We understand the concerns of the Reviewer regarding some overly optimistic generalizations in the manuscript. Thus, we have tampered down all relevant passages in the manuscript.

We demonstrate that visual information in our sequential task is stored in independent pools of contrasting resources, with perfect and imperfect retrieval rates.

How does our brain store and retrieve visual information when we receive it piece-by-piece, rather than all at once? Here, we demonstrate how a detailed computational model of visuospatial working memory (VSWM) offers a clear answer to this question in the context of a task widely used in clinical research.

Our modeling clearly demonstrates that in this sequential visuospatial memory task, participants make use of different resources with dissimilar properties.

With the goal of characterizing the mechanisms underlying this sequential memory task, here we developed a family of computational models of how information is progressively stored and retrieved from memory in Owen’s sequential task.

Our work adds to this tradition by formalizing and demonstrating separate stores of differential reliability and capacity for items in Owen’s sequential working memory task. In other words, sequential visuospatial information is retained in several ‘compartments’.

### Comment 3

*I would also like to see more discussion around assumption 6, described on page 8, that “Participants select items from the decision pool randomly.” If I were to complete the task, I think I would try to employ a systematic search strategy so as to minimise the within-search memory load, and focus on trying to remember between–search locations. Is there evidence for participants employing such a strategy?*

We thank the Reviewer for this question that brings to light a subtle but important aspect of the model: It does not predict which specific item a participant will tap, but rather, the probability that the next tap will be a correct choice or an error, and which type of error. We acknowledge that it is fundamental limitation of our model, that it can’t structurally predict the probability that a participant will pick a specific item. Since the model can’t make this level of predictions, we assume that participants pick items randomly.

We clarified this point in the *Methods* and *Discussion* sections.

Here, it is important to note that with this approach we seek to predict the probability that a participant will make an error, and which type of error, but not the probability that a specific item is selected. This limitation of the model is captured by the assumption that items are selected randomly from the decision pool, as the model does not predict which specific item will be tapped. Non-random strategies like, for example, first select items from the top of the screen, are fundamentally compatible with our approach, as they provide a further level of granularity that we don’t seek to model here.

Several aspects of how participants solve Owen’s task are not captured in our framework. For example, already (Owen et al., 1990) recognized that participants tend to start searches from the same item and follow stereotypical patterns while tapping items that they believed are empty. The ability to follow this strategy has been suggested to reflect ‘executive’ control, rather than memory capacity (Owen et al., 1997). Other aspects of the task that are also not covered by the model are recency effects on between-search items, whereby last correctly tapped items are less likely to lead to errors. In general, the models proposed here do not predict which specific items are tapped, but what is the probability that the next tap of a participant will be an error. Thus, our model cannot capture phenomena like ‘strategic’ searching and the recency effect. While this feature of the model seems to have a limited effect on the ability of the model to predict errors in the task, this could explain small but systematic biases in the predictions of the model, as observed in Fig. 3A and Supp. Fig. 8.

### Comment 4:

*Page 33 – the CANTAB Spatial Working Memory task is described. Within-search constraints are described well, but I would like to see more information about between-search constraints.*

We have further clarified the structure of the CANTAB Spatial Working Memory task.

The CANTAB Spatial Working Memory Component (CSWMC) consisted of two training rounds of set size 4, followed by six games of set size 4, 6 and 8 items. The target (a yellow square) was randomly hidden behind one of the items (a different color square). As an additional rule, after a participant had searched two empty items, the location of the target was modified to be the next empty item searched. Thus, participants never had to remember more than two within-search items, reducing the length and complexity of the task. In contrast to FtE, there was no limit on the number of WSE and BSE so that a game only stopped after all targets were found. No further specific constraint applied to between-search items.

## Reviewer #2

### Comment 1

*I would like to congratulate the authors for this interesting study. This work helps us understand how the brain stores and retrieves visual information and shows that a model that includes stochastic retrieval of information outperforms the models without, at least in people with ASD and AD. In addition the authors show that their computational modelling could be a reliable measure of cognitive function using data from three different clinical trials. I only have one major comment, a few clarifications and some discussion points. Thus overall I think the paper is strong.*

We thank the Reviewer for the supportive words.

### Comment 2

*My only concern is that the question of the paper according to the author's summary is “how does the brain store and retrieve visual information when we receive it piece by piece, rather than all at once?” To understand this question the authors should collect data with healthy participants and then study what are the differences with the different clinical populations.*

We acknowledge that a limitation of this study is the comparatively small amount of data from neurotypical participants. A large fraction of the data used in our manuscript was collected in the context of clinical trials that by design do not include neurotypical participants. Nevertheless, 33 control participants were included in oRBiting. We briefly acknowledge this limitation in the discussion section.

Most participants in the three studies considered here were clinically diagnosed with autism or MCI/early AD, with only a small minority being considered neurotypicals. While the relative model probability did not differ significantly between ASD and neurotypicals, futures studies are needed to confirm and extend this initial observation

Interestingly, when we compared the model evidence of the different models we tested across the ASD and neurotypical groups, we did not find any differences between the two groups or between age groups, suggesting no large difference in the fit of the models across groups.

We also calculated the relative probability of each model on the individual participant level. The mean relative probability of a model across participants represents the expected fraction of individuals in which that model is favored. Overall, the full model was favored by between 61 and 65% of participants, with the model without deterministic storage being favored by between 29 and 34% of participants. Thus, most participants used some form of non-deterministic retrieval, and the majority also relied on perfectly reliable storage.

To investigate if there were differences between the models across groups in oRBiting, we applied MANOVA on the participants’ model probabilities transformed to the real interval. Participants with IQ below 70 were excluded, as this group was composed exclusively of autistic people. We found no effect of diagnostic group (P=0.15), or age (P=0.92).

### Comment 3

*This might be a matter of style but sometimes I would like some more information about the methods in the main text. For instance in lines 257-260, it is explained that the model was fitted. Although the parameters that are actually fitted are in the methods section, I think a brief explanation of how the model was fitted and what were the actual fitted parameters will help the reader to follow the paper.*

As suggested by the Reviewer, we have added a short explanation of the methods used to fit the model in the *Results*, to increase the clarity of the manuscript.

Models were fitted quantitatively using the Hamiltonian Monte Carlo (HMC) algorithm as implemented in TensorFlow Probability (Dillon et al., 2017). HMC is a hybrid algorithm that uses the gradient of a target distribution to efficiently sample from it. The gradient computation is automatically handled by TensorFlow, using automatic differentiation. The target probability of the models is the trial-by-trial likelihood multiplied by the prior probability of the model parameters, as specified in the *Methods* section. The full model has two discrete parameters M_B and M_stoch, which could range from 0 to the largest set size possible in the task in addition to two continuous parameters: the probability of a slip η and the retrieval rate r. Since the HMC algorithm can only be used on continuous parameters, the discrete parameters can be easily marginalized out by computing

Once samples from the marginal posterior probability of parameters *eta* and *r* were drawn using the HMC algorithm, these were used to estimate M_total and to make posterior model predictions. Data from all sessions of a single participant were pooled together to obtain individual posterior estimates that were used to predict participant’s response on a trial-by-trial basis. Details of the inference are presented in the *Methods* and the code used for model fitting is openly available at<https://osf.io/3ufg5>.

### Comment 4

*Similarly the hierarchical model could also be briefly explained in the results section. Actually, I do not really understand why you need this type of model. Would it not be equivalent to fit the data separately for different sessions and then study the Mtotal across sessions? I would expect that early sessions will find higher Mtotal.*

As suggested by the Reviewer, it is entirely possible to fit the model to individual sessions and to compare the estimates from each session. Similar to a random effects model, the hierarchical model was introduced to exploit the correlations among data from different sessions from a single participant. In our case, this is particularly important because the small amount of data in each session renders the model estimation challenging.

Regarding the observation that M_total might change over time, our data from AD patients indicates that after an uptick following the Screening session, it declined over the course of the next two years. Interestingly, in the shorter studies in ASD participants, we did not observe any change in M_total (as shown in the new Supp. Fig. 10, displayed below).

Following an uptick in M_total after the screening session—likely due to familiarization with the test—there was a decline in M_total over the next two years (Fig. 6; days in CT: t_345=-2.5,P=0.011).

We now explain in the manuscript the reason to introduce a hierarchical model to estimate *M_total*.

Given the longitudinal nature of our question, we extended the model to capture changes in working memory using a hierarchical approach, explained in detail in the Methods section. This hierarchical approach is essentially a random effects model that captures the correlations between sessions from a single individual while allowing for changes across sessions. Since the data from any individual session is limited, this extension of the model improves our ability to capture longitudinal changes in memory capacity.

### Comment 5

*Table 1: When comparing different models with AIC or BIC, I found it more useful when reported as de ΔAIC with respect to the models that best described the data.*

We thank the reviewer for this suggestion, which we have implemented.

### Comment 6

*Fig 5: Correlation between IQ and memory capacity seems rather low. I think the statement about the correlation between IQ and Mtotal should be turned down. Could the authors quantify the magnitude of the correlation? What is r-square? Seems that the IQ is more correlated to the Mtotal for V1aduct than ORBITING. Is there any reason for that? Was the V1aduct evaluated in the lab versus smartphone for oRBiting?*

We thank the Reviewer for this thoughtful comment that prompted us to revisit the scripts generating Fig. 5. We noted that the script plotted data from a previous version of the model as the one used for the rest of the results in the manuscript. We have updated the figure to represent the correct version of the model. This does not bear any consequence on the overall interpretation of the findings.

As the Reviewer suggests, the correlation between M_total and IQ is stronger in V1aduct (*r=0.58, R2=0.33)* compared to oRBiting (r=0.32, *R2=0.10*). There are a number of possible explanations for this. As we noted in the manuscript, some individuals with IQ over 120 in oRBiting performed particularly poorly in the task, at a level similar to individuals with IQ below 60, suggesting poor compliance. More interestingly, we suspect a floor effect in the memory capacity metric in individuals with IQ below 70. However, this also highlights that participants in V1aduct were all autistic adults with IQ above 70, whereas oRBiting included children and adolescents, with and without intellectual disability. This difference in the cohorts partly explains the difference in the correlation coefficients, as most of the error in oRBiting seems to originate from participants with poor performance in the task and low IQ, as shown below.

Directly answering one of the questions of the Reviewer, in both studies participants engaged with FtE at home.

Regarding the magnitude of the correlation coefficients between IQ and our index of working memory, an influential metanalysis based mostly on studies conducted on college students found that “on average (weighted by sample size), working memory tests correlated .364 with measures of *g*. Correcting for unreliability of working memory and *g* measures increased the average correlation between WM and g to .479, yielding a shared variance of 22.9%.” [(1)](https://www.zotero.org/google-docs/?xKlwQG). Thus, the *r* values reported here are within reasonable expectations from previous literature.

We expanded on our discussion of the differences between both studies.

A handful of oRBiting’s participants with IQ close or above 120 performed poorly in the task, suggesting compliance issues that can be expected from a smartphone assessment. Importantly, oRBiting included individuals with IQ below 70, usually considered a sign of intellectual disability, for whom FtE might be less appropriate.

### Comment 7

*It could be interesting to discuss if FtE could be used not only in clinical trials to track cognitive function but also as a biomarker for diseases. For instance, we could just play the game on our smartphones regularly to detect reductions in Mtotal that are larger than expected and indicative of some sort of early dementia or AD.*

We thank the Reviewer for raising this interesting point. Indeed, we believe that FtE together with other remote digital tests could be helpful in identifying cognitive impairments in pre-manifest AD. Some recent literature suggests similar applications, which we elaborate on in the discussion.

Beyond its application as a digital/computational biomarker of disease progression in AD, it might be possible to use this approach to screen for the subtle changes in cognition that characterize mild cognitive impairment. This type of biomarkers could serve as a scalable and inexpensive approach to gate more in-depth or invasive diagnostic procedures, such as clinical cognitive testing or amyloid PET (Tideman et al., 2025; Tröger et al., 2022). It remains an open question whether computational approaches that model the cognitive processes behind measurable behavior will indeed improve the sensitivity and specificity of digital biomarkers of cognition.

### Comment 8

*For discussion, I agree with the authors that quantitative measures of cognitive function are necessary to better evaluate clinical trials because we can detect and quantify the potential changes much better. However, I also think it is important to relate those changes to changes in the quality of life of patients. In other words, it is great that we can detect a change in 0.1 items in Mtotal, but what does this mean for the patient? Is their quality of life reduced by that? I think that if we want to use these quantitative measures in clinical trials to show efficacy, we will have to work on what they mean to the patients.*

The Reviewer raises a very important matter that has deep ramifications for how we approach the development of treatments for diseases like AD. There are two angles to it - one is to consider that biomarkers of cognition, while important to detect treatment effects or worsening of a disease, are not truly meaningful to patients. Indeed, visuospatial working memory is unlikely to be a meaningful construct for most individuals with neurodegenerative diseases and their caregivers. From a second angle, it is increasingly recognized that subtle cognitive changes that occur early in disease precede true impairments in patients’ daily lives. It is now recognized by the [FDA](https://www.fda.gov/media/110903/download) that to develop preventive treatments targeting pre-manifest AD, it will be paramount to measure cognitive changes even before they affect participants' daily life.

We found that our index of working memory M_total is correlated with the CDR SOB, an end point accepted by regulators (Fig. 6G). However, from this figure, it is clear that M_total is far more variable than CDR SOB. Note however, that M_total was computed from infrequent, in-clinic sessions. Future research will be needed to understand the sensitivity and overall psychometric properties of FtE. We briefly discuss this topic in the manuscript.

M_total was associated with both cognitive and functional endpoints (ADAS-Cog 13, CDR-SOB) in AD, indicating that, despite possible limitations in its psychometric properties, it reflects constructs that could matter to patients and their caregivers. Further work will be needed to explore these associations in AD and other indications.

## Reviewer #3

### Comment 1 & 2

*In this work, the authors investigate how visuospatial information is stored and retrieved when stimuli are presented sequentially rather than simultaneously, using a computational model applied to behavioral data from three human cohorts. A major strength is the inclusion of neurotypical, autistic, and prodromal Alzheimer’s participants, which allows testing the robustness of the model across populations and task implementations. The results support a multi-store account of sequential visuospatial working memory with distinct retrieval properties. While the modeling approach is compelling, several assumptions and observed behavioral results warrant further clarification. Given the richness of the datasets, some aspects could be further explored to strengthen the conclusions. Overall, I recommend the study for the PLOS Computational Biology audience pending some major revisions.*
Major comments
*1. The authors carefully assess errors related to memory encoding and retrieval, but there is a critical absence of consideration for memory maintenance. Sequential tasks inherently involve maintaining information over time, and neglecting this aspect could confound interpretations of memory capacity. It would strengthen the study to either expand the modeling framework to include maintenance dynamics or provide a thorough assessment of how maintenance might or not be influencing the results. Some of the datasets may already contain the temporal structure necessary to explore this, potentially allowing for modeling beyond simple binary parameters and providing richer insight into participants’ memory errors.*

*2. Expanding on the first point more generally, some of the assumptions in this work may be conflating capacity and retrieval reliability with strategy changes and task engagement. It would strengthen the manuscript to more explicitly discuss these potential confounds and alternative explanations for the observed error patterns, particularly given the absence of direct measures of underlying cognitive or neural processes. For example, more in-depth characterization of response time patterns and within session changes.*

We thank the Reviewer for this insightful comment, which triggered us to assess the impact of not accounting for maintenance on our analysis. We examined the root mean square error (RMSE) of the predicted error rate against the actual error rate as a function of the trial number in a game. Higher trial number is a proxy measure of how long a participant had maintained information during a given game. If the RMSE increases with the trial number, this would indicate that the number of trials is a factor that the model cannot account for and that plays a role in participants’ behavior. In the new Supp. Figure 7 displayed below, on the left we plot first the percentage of trials as a function of trial number and set size and on the right the RMSE of the model-based predictions as a function trial number.

Supp. Figure 7: A) Distribution of the trials as a function of set size and trial number in game. Trial number is proxy measure of memory maintenance, as it is an index of the duration of the game up to that specific trial. B) Root mean square error (RMSE) of the predicted error rate by the full model and the observed error rate as a function trial number. The RMSE clearly increases with trial number, indicating that memory maintenance plays a role in participants’ behavior that remains unexplained by the model.

This figure suggests that maintenance indeed plays a role that is not accounted for by the model. This is especially true for trials that require long memory maintenance, with the caveat that these constitute a small fraction of the data, as shown by the left panel. As expected, these trials belong mostly to the condition with the largest set size. This matches with the Reviewer’s observation regarding the quality of the fist in this condition, in which the model consistently underestimates participants’ error rate. We added this to the limitations of the model in the *Discussion* (text shown below).

Another factor not evaluated in the manuscript is primacy and recency effects, that is, whether the first and last tapped between-search items are less likely to be associated with errors than predicted by chance. As we discussed above, in its current form, the model does not predict which particular item will be searched by a participant, and thereby it can’t account for temporal effects as recency and primacy. In the figure below, we explored these two possibilities.

In the absence of a recency or primary effects, one would expect that the first and last between-search items in a game would lead to errors at chance (green line), that is, with a probability of 1/# between-search items. However, the last between-search item is associated with fewer errors than expected by chance, suggesting a recency effect. Interestingly, the opposite of the primacy effect is observed when few between search items are present but this effect disappears at higher loads. Thus, there is no evidence of primacy in this task.

Overall, as noted by the Reviewer, several aspects of the task remain unexplored. We discuss this now in the manuscript.

Several aspects of how participants solve Owen’s task are not captured in our framework. For example, already (Owen et al., 1990) recognized that participants tend to start searches from the same item and follow stereotypical patterns while tapping items that they believed are empty. This strategy is considered to reflect ‘executive control’, rather than memory capacity (Owen et al., 1997).

A further factor not integrated into the current version of our model is the limitation in participance’s maintenance of information over the course of the task. This factor is likely to account for the consistent underestimation of participant’s error rate in trials that require particularly high maintenance (Supp. Fig. 7). Other aspects of the task that are also not covered by the model are recency effects on between-search items, whereby the last correctly tapped item is less likely to lead to an error.

In general, the models proposed here do not predict which specific items are tapped, but what is the probability that the next tap of a participant will be an error. Thus, our model cannot capture phenomena like ‘strategic’ searching, and the recency effect. While this feature of the model seems to have a limited effect on the ability of the model to predict errors in the task, this could explain small but systematic biases in the predictions of the model, as observed in Fig. 3A and Supp. Fig. 8.

### Comment 3

*3. The authors report very low within-search errors (WSE) across most conditions and therefore fix within-search capacity to exceed the maximum set size. However, at the highest loads, WSE increases sharply with the number of between-search items, deviating from model predictions. This suggests that the assumption of independence between within- and between-search performance may break down under extreme demands. The authors should provide a more thorough discussion of whether this reflects a limitation of the current model, additional cognitive constraints, or properties of the task design itself. Relatedly, clarifying why WSE is so low overall and whether this was expected or consistent across groups (including AD participants), would help interpret the model’s relevance.*

Regarding the first question of the Reviewer, we consider the sharp increase in WSE at high loads a true deviation from the predictions of our model, as information from between-search items interfere with the storage of within-search items. While the model could be potentially modified to account for this deviation, these errors constitute a minute fraction of our data and are probably not amenable for modeling given the current design of the task. However, they constitute an interesting venue for future research. We discuss this now in the manuscript.

Very low WSE is typical for both FtE and in the CANTAB spatial memory task. For this reason, WSE are rarely carefully analyzed or reported, as the dynamic range of this measure is limited. Our approach, which seems to capture a good fraction of these errors, suggests that at low load conditions, these are mostly true slips, which can be expected especially in unsupervised settings.

Interestingly, we found that at very high loads, the number of WSE sharply increases with the number of between-search items. This constitutes a true violation of the assumption of two independent stores. This could indicate that there is no complete differentiation between the two memory stores or that there is some flexibility on how resources are allocated depending on the difficulty of the task. Unfortunately, these errors constitute a very small fraction of the data precluding their rigorous analysis.

### Comment 4

*4. The model version that was used to fit data for AD patients includes an extension that copes with time dependent variation. It is unclear why this expanded model was not applied to the other datasets to explore whether similar dynamics occur in neurotypical or autistic participants. Applying it more broadly could help establish the generality of the approach and clarify whether capacity and retrieval parameters are stable across time in different populations.*

Following the suggestion of the Reviewer, in the supplemental material we now include a longitudinal analysis of the data from both oRBiting and V1aduct using the hierarchical model. We found no evidence of a change in time in the total memory using a mixed effects model including random effects for the base line and rate of change of each participant as shown below.

**Supp. Figure 10: A & B)** Estimated total memory capacity in oRBiting (A) and V1aduct (B) as a function of session. Faded lines correspond to individual estimates, solid black lines to the estimated marginal effect of session.

### Comment 5

*1. Given the addition of autistic patients, it would be interesting to mention whether there were any differences between those and the neurotypical subjects.*

Since oRBiting was not designed to identify differences between people with autism and neurotypicals, we did not consider reporting this analysis as appropriate as it could potentially stigmatize people with autism. We report the findings here, but we don’t include them in the manuscript.

Since the study did not include a neurotypical group with IQ below 70, we removed the AD low IQ group from this statistical analysis. Using a model including age and gender as covariates, we found no statistically significant difference between the ASD and neurotypical group in total working memory (Diff neurotypical vs ASD = 0.7, SD 0.64, t=1.099, P=0.275).

### Comment 6

*One of the main figures for the first part of the paper (Fig. 3) shows the fit for one dataset, while the second dataset is pushed to Supplementary material. It would be clearer to provide a combined plot for both datasets or alternatively explain the rationale for choosing one or the other as a main figure.*

Given the clear similarities between both datasets, we considered it uninformative to include both datasets and fits in the main manuscript. However, for an interested reader, we found it reassuring to also show the data from V1aduct (not displayed in the main manuscript), demonstrating the similarities between participants’ behavior and model fits across the two studies.

Following the suggestion of the Reviewer, we now also include a figure (Supp. Fig. 5) with the pooled datasets in the Supplementary Materials. This is mentioned now in the manuscript.

Qualitatively, we closely replicated our findings in V1aduct, as shown in Supp. Fig. 4. Supp. Fig. 5 displays a pooled analysis of both studies.

### Comment 7

*The table (Table 1) depicting the model comparison seems a bit simple despite supporting one of the main claims of the paper (the need for both stochastic and deterministic retrieval). Is the full model better across the board for all subjects and sessions? Do differences in model fitting depend on the load? Expanding on the model comparison would make a more convincing point about the need for the full model.*

To address this comment, we used a simplified version of a statistical approach commonly used to compare models across participants [(2)](https://www.zotero.org/google-docs/?y4lQtb). Every model is compared at a subject level using the normalized model evidence (defined as -2 WAIC), yielding a probability between 0 and 1 for each model, such that the sum of the probabilities of all models is equal to 1. A high value indicates that a model is favored in an individual participant. We report the mean model evidence across participants on each cohort, as shown below.

As can be seen, the expected model probabilities favor clearly the full model, in both data sets in around 60% of participants. The second most favored model was M_stoch, which includes stochastic storage. We include this analysis in the manuscript.

Regarding nonstationary of the model, while an important possibility, investigating it would require a much more complex model that allows for within-session changes in the parameters of the model.

### Comment 8

*Despite the qualitatively good fitting of the model to the data, there is a systematic drift where the model slope is steeper than the empirical data at smaller set sizes and flatter at larger set sizes. The authors should provide some logic that helps explain this drift as it is regularly present in all the datasets used.*

In our reply to comments 1 & 2 of Reviewer 3 we provide a more detailed discussion of the limitations of the model and possible explanations for the small biases of the model fits.

### Comment 9

*While Supplementary Figure 2 provides some details about the behavioral results, it would be beneficial to expand this information to all datasets. Additionally, including other temporal metrics such as breaks between blocks, trial durations, and response times could help interpret some of the results.*

Unfortunately, FtE was not designed to measure reaction times (RT) and no rigorous information on participants RT was collected. In the two studies here, only time stamps indicating the beginning of a trial were collected. Moreover, correct trials in which participants found a target (egg) had a longer duration because the target was displayed and participants were asked to drag it to the basket on the bottom of the screen. Thus, we consider it inadequate to include this analysis in the main manuscript, but we have included it here for transparency. In addition, we expand on other behavioral analysis in the Supp. Table 2.

Trial length in A) oRBiting and B) V1aduct. The length of the trial corresponds to the time elapsed between the start and end of a trial, and thus, it includes the intertrial interval. BSE: Between-search errors. WSE: Within-search errors. ms: Milliseconds.

Except for correct trials with a target, all other trials were not significantly different in their duration. For the case of oRBiting, using a mixed effect model with participant as random effect, and trial type, age, sex, and diagnosis as fixed effects, we found that only trial type had a significant effect (P<10^-5^) on trial length. The same pattern was observed in V1aduct.

We now include summary statistics of key behavioral metrics in the Supp. Table 2.

### Comment 10

*1. Figure 1 schematic could be improved for further clarity as the concepts between and within search are crucial for the rest of the work. The difference between the two is somewhat unclear.*

We have improved Figure 1 for clarity.

### Comment 11

*2. Figure 6 is small and hard to read. Some of the terminology (Schematics and Baseline) should be consistently capitalized, and methods should clarify what these refer to.*

We have improved the visual presentation of this figure and added an explanation for each abbreviation.

### Comment 12

*3. In line 160, the correct reference should be Supp. Fig. 1.*

We have corrected this in the text now.

In Supp. Text 2 and Supp. Fig. 1, we present two variations of this model in which between-search items are stored before all within-search items or vice versa.
