# Supplementary material for: Beyond memory capacity: A probabilistic, dual store model of visuospatial working memory
Source: PLoS Comput Biol. 2026 Jul 20;22(7):e1014535. doi: 10.1371/journal.pcbi.1014535 (PMC13475967; doi:10.1371/journal.pcbi.1014535)
Supplement: Peer review history

# Peer review: Decision Letter 0

**DOI:** 10.1371/journal.pcbi.1014535.r001

**Authors:** Tianming Yang, Jorge Mejias

16 Feb 2026

Beyond memory capacity: A probabilistic, dual store model of visuospatial working memory

PLOS Computational Biology

Dear Dr. Aponte,

Thank you for submitting your manuscript to PLOS Computational Biology. After careful consideration, we feel that it has merit but does not fully meet PLOS Computational Biology's publication criteria as it currently stands. Therefore, we invite you to submit a revised version of the manuscript that addresses the points raised during the review process.

Please submit your revised manuscript by Apr 18 2026 11:59PM. If you will need more time than this to complete your revisions, please reply to this message or contact the journal office at ploscompbiol@plos.org. When you're ready to submit your revision, log on to https://www.editorialmanager.com/pcompbiol/ and select the 'Submissions Needing Revision' folder to locate your manuscript file.

Please include the following items when submitting your revised manuscript:

* A letter that responds to each point raised by the editor and reviewer(s). You should upload this letter as a separate file labeled 'Response to Reviewers'. This file does not need to include responses to formatting updates and technical items listed in the 'Journal Requirements' section below.

* A marked-up copy of your manuscript that highlights changes made to the original version. You should upload this as a separate file labeled 'Revised Manuscript with Track Changes'.

* An unmarked version of your revised paper without tracked changes. You should upload this as a separate file labeled 'Manuscript'.

If you would like to make changes to your financial disclosure, competing interests statement, or data availability statement, please make these updates within the submission form at the time of resubmission. Guidelines for resubmitting your figure files are available below the reviewer comments at the end of this letter

We look forward to receiving your revised manuscript.

Kind regards,

Jorge F. Mejias, Ph.D.

Academic Editor

PLOS Computational Biology

Joseph Ayers

Section Editor

PLOS Computational Biology

**Additional Editor Comments:**

All three reviewers found the work interesting and potentially suitable for publication in PLOS Computational Biology. They also identified several points that should be addressed in the revision, including some issues in the presentation of of the results and refinement of some claims, the absence of details in the methods and results section, and a more comprehensive assessment of some of the results. Please keep in mind that the code to replicate the model results should also be made available as per the PLOS Data Policy guidelines.

**Journal Requirements:**

If the reviewer comments include a recommendation to cite specific previously published works, please review and evaluate these publications to determine whether they are relevant and should be cited. There is no requirement to cite these works unless the editor has indicated otherwise.

1) Please ensure that the CRediT author contributions listed for every co-author are completed accurately and in full.

At this stage, the following Authors/Authors require contributions: Christopher H Chatham, Francesca Cormack, and Thanneer M Perumal. Please ensure that the full contributions of each author are acknowledged in the "Add/Edit/Remove Authors" section of our submission form.

The list of CRediT author contributions may be found here: https://journals.plos.org/ploscompbiol/s/authorship#loc-author-contributions

2) Please upload all main figures as separate Figure files in .tif or .eps format. For more information about how to convert and format your figure files please see our guidelines:

https://journals.plos.org/ploscompbiol/s/figures

3) We have noticed that you have uploaded Supporting Information files, but you have not included a list of legends. Please add a full list of legends for your Supporting Information files after the references list.

4) Some material included in your submission may be copyrighted. According to PLOSu2019s copyright policy, authors who use figures or other material (e.g., graphics, clipart, maps) from another author or copyright holder must demonstrate or obtain permission to publish this material under the Creative Commons Attribution 4.0 International (CC BY 4.0) License used by PLOS journals. Please closely review the details of PLOSu2019s copyright requirements here: PLOS Licenses and Copyright. If you need to request permissions from a copyright holder, you may use PLOS's Copyright Content Permission form.

Please respond directly to this email and provide any known details concerning your material's license terms and permissions required for reuse, even if you have not yet obtained copyright permissions or are unsure of your material's copyright compatibility. Once you have responded and addressed all other outstanding technical requirements, you may resubmit your manuscript within Editorial Manager.

Potential Copyright Issues:

i) Figure 1. Please confirm whether you drew the images / clip-art within the figure panels by hand. If you did not draw the images, please provide (a) a link to the source of the images or icons and their license / terms of use; or (b) written permission from the copyright holder to publish the images or icons under our CC BY 4.0 license. Alternatively, you may replace the images with open source alternatives. See these open source resources you may use to replace images / clip-art:

- https://commons.wikimedia.org

- https://openclipart.org/.

5) We note that you have indicated that there are restrictions to data sharing for this study. PLOS only allows data to be available upon request if there are legal or ethical restrictions on sharing data publicly. For more information on unacceptable data access restrictions, please see https://journals.plos.org/ploscompbiol/s/data-availability#loc-unacceptable-data-access-restrictions.

Before we proceed with your manuscript, please address the following prompts:

a) If there are ethical or legal restrictions on sharing a de-identified data set, please explain them in detail (e.g., data contain potentially identifying or sensitive patient information, data are owned by a third-party organization, etc.) and who has imposed them (e.g., a Research Ethics Committee or Institutional Review Board, etc.). Please also provide contact information for a data access committee, ethics committee, or other institutional body to which data requests may be sent.

b) If there are no restrictions, please upload the minimal anonymized data set necessary to replicate your study findings to a stable, public repository and provide us with the relevant URLs, DOIs, or accession numbers. For a list of recommended repositories, please see https://journals.plos.org/plosone/s/recommended-repositories. You also have the option of uploading the data as Supporting Information files, but we would recommend depositing data directly to a data repository if possible.

We will update your Data Availability statement on your behalf to reflect the information you provide.

6) Please amend your detailed Financial Disclosure statement. This is published with the article. It must therefore be completed in full sentences and contain the exact wording you wish to be published.

1) State the initials, alongside each funding source, of each author to receive each grant. For example: "This work was supported by the National Institutes of Health (####### to AM; ###### to CJ) and the National Science Foundation (###### to AM)."

2) State what role the funders took in the study. If the funders had no role in your study, please state: "The funders had no role in study design, data collection and analysis, decision to publish, or preparation of the manuscript."

3) If any authors received a salary from any of your funders, please state which authors and which funders..

If you did not receive any funding for this study, please simply state: u201cThe authors received no specific funding for this work.u201d

7) Your current Financial Disclosure states, "Yes ↳ Please add funding details. This research was solely funded by F. Hoffmann-La Roche Ltd. ↳ Please select the country of your main research funder (please select carefully as in some cases this is used in fee calculation). SWITZERLAND - CH".

However, your funding information on the submission form indicates no funds.

Please indicate by return email the full and correct funding information for your study and confirm the order in which funding contributions should appear. Please be sure to indicate whether the funders played any role in the study design, data collection and analysis, decision to publish, or preparation of the manuscript.

8) Please send a completed 'Competing Interests' statement, including any COIs declared by your co-authors. If you have no competing interests to declare, please state "The authors have declared that no competing interests exist". Otherwise please declare all competing interests beginning with the statement "I have read the journal's policy and the authors of this manuscript have the following competing interests"

**Reviewers' comments:**

Reviewer's Responses to Questions

**Comments to the Authors:**

**Please note here if the review is uploaded as an attachment.**

Reviewer #1: I believe that the results presented here are interesting and useful. The authors have used a computational modelling approach to capture the pattern of errors seen in the “Fing the Egg” task and CANTAB Spatial Working Memory task. Importantly, both of these tasks require participants to search between items while holding in memory information about targets during previous searches (between search items) and also information about items previously searched within the current search (within search items). A modelling approach that accounts for these two types of storage, as well as allowing for separate storage v retrieval errors, provided the best account across two datasets.

My main concern relates to how these results are presented. The abstract describes how the model explains how visual information is stored “in sequential tasks”. The Authors Summary asks “how does our brain store and retrieve visual information when we receive it piece-by-piece, rather than all at once?” The introduction describes how “our limited understanding of how sequentially acquired information is processed in VSWM represents a critical gap”. I believe this is misleading. The authors have successfully modelled one very specific type of sequential task; one that involves a search task and the need to retain both within and between search information. Arguably, most sequential VSWM tasks do not involve this requirement. Instead, participants are asked to remember independent sequences of items (eg. the Corsi block task), or monitor a stream of sequentially presented items (eg. a visuo-spatial n-back task). The multi-store modelling approach presented here can not be used for tasks such as these, because they do not involve remembering within as well as between search/session items.

Statements like this “We demonstrate that visual information in sequential tasks is stored in a variety of ways that impose contrasting constraints, showing the existence of pools of independent resources, with perfect and imperfect retrieval rates” (abstract) and “In summary, our qualitative analysis showed that sequential recalling of items is mediated 288 by two types of memory: one for within-search items and one for between-search items” (page 20, line 287) are particularly misleading, as the results only relate to a specific type of sequential task, not sequential tasks in general.

I would also like to see more discussion around assumption 6, described on page 8, that “Participants select items from the decision pool randomly.” If I were to complete the task, I think I would try to employ a systematic search strategy so as to minimise the within-search memory load, and focus on trying to remember between–search locations. Is there evidence for participants employing such a strategy?

Page 33 – the CANTAB Spatial Working Memory task is described. Within-search constraints are described well, but I would like to see more information about between-search constraints.

Reviewer #2: I would like to congratulate the authors for this interesting study. This work helps us understand how the brain stores and retrieves visual information and shows that a model that includes stochastic retrieval of information outperforms the models without, at least in people with ASD and AD. In addition the authors show that their computational modelling could be a reliable measure of cognitive function using data from three different clinical trials. I only have one major comment, a few clarifications and some discussion points. Thus overall I think the paper is strong.

My only concern is that the question of the paper according to the author's summary is “how does the brain store and retrieve visual information when we receive it piece by piece, rather than all at once?” To understand this question the authors should collect data with healthy participants and then study what are the differences with the different clinical populations.

Minor comments

This might be a matter of style but sometimes I would like some more information about the methods in the main text. For instance in lines 257-260, it is explained that the model was fitted. Although the parameters that are actually fitted are in the methods section, I think a brief explanation of how the model was fitted and what were the actual fitted parameters will help the reader to follow the paper.

Similarly the hierarchical model could also be briefly explained in the results section. Actually, I do not really understand why you need this type of model. Would it not be equivalent to fit the data separately for different sessions and then study the Mtotal across sessions? I would expect that early sessions will find higher Mtotals.

Table 1: When comparing different models with AIC or BIC, I found it more useful when reported as de ΔAIC with respect to the models that best described the data.

Fig 5: Correlation between IQ and memory capacity seems rather low. I think the statement about the correlation between IQ and Mtotal should be turned down. Could the authors quantify the magnitude of the correlation? What is r-square? Seems that the IQ is more correlated to the Mtotal for V1aduct than ORBITING. Is there any reason for that? Was the V1aduct evaluated in the lab versus smartphone for oRBiting?

It could be interesting to discuss if FtE could be used not only in clinical trials to track cognitive function but also as a biomarker for diseases. For instance, we could just play the game on our smartphones regularly to detect reductions in Mtotal that are larger than expected and indicative of some sort of early dementia or AD. Do you think that's possible?

For discussion, I agree with the authors that quantitative measures of cognitive function are necessary to better evaluate clinical trials because we can detect and quantify the potential changes much better. However, I also think it is important to relate those changes to changes in the quality of life of patients. In other words, it is great that we can detect a change in 0.1 items in Mtotal, but what does this mean for the patient? Is their quality of life reduced by that? I think that if we want to use these quantitative measures in clinical trials to show efficacy, we will have to work on what they mean to the patients.

Reviewer #3: Summary

In this work, the authors investigate how visuospatial information is stored and retrieved when stimuli are presented sequentially rather than simultaneously, using a computational model applied to behavioral data from three human cohorts. A major strength is the inclusion of neurotypical, autistic, and prodromal Alzheimer’s participants, which allows testing the robustness of the model across populations and task implementations. The results support a multi-store account of sequential visuospatial working memory with distinct retrieval properties. While the modeling approach is compelling, several assumptions and observed behavioral results warrant further clarification. Given the richness of the datasets, some aspects could be further explored to strengthen the conclusions. Overall, I recommend the study for the PLOS Computational Biology audience pending some major revisions.

Major comments

1. The authors carefully assess errors related to memory encoding and retrieval, but there is a critical absence of consideration for memory maintenance. Sequential tasks inherently involve maintaining information over time, and neglecting this aspect could confound interpretations of memory capacity. It would strengthen the study to either expand the modeling framework to include maintenance dynamics or provide a thorough assessment of how maintenance might or not be influencing the results. Some of the datasets may already contain the temporal structure necessary to explore this, potentially allowing for modeling beyond simple binary parameters and providing richer insight into participants’ memory errors.

2. Expanding on the first point more generally, some of the assumptions in this work may be conflating capacity and retrieval reliability with strategy changes and task engagement. It would strengthen the manuscript to more explicitly discuss these potential confounds and alternative explanations for the observed error patterns, particularly given the absence of direct measures of underlying cognitive or neural processes. For example, more in-depth characterization of response time patterns and within session changes.

3. The authors report very low within-search errors (WSE) across most conditions and therefore fix within-search capacity to exceed the maximum set size. However, at the highest loads, WSE increases sharply with the number of between-search items, deviating from model predictions. This suggests that the assumption of independence between within- and between-search performance may break down under extreme demands. The authors should provide a more thorough discussion of whether this reflects a limitation of the current model, additional cognitive constraints, or properties of the task design itself. Relatedly, clarifying why WSE is so low overall and whether this was expected or consistent across groups (including AD participants), would help interpret the model’s relevance.

4. The model version that was used to fit data for AD patients includes an extension that copes with time dependent variation. It is unclear why this expanded model was not applied to the other datasets to explore whether similar dynamics occur in neurotypical or autistic participants. Applying it more broadly could help establish the generality of the approach and clarify whether capacity and retrieval parameters are stable across time in different populations.

Minor comments

1. Given the addition of autistic patients, it would be interesting to mention whether there were any differences between those and the neurotypical subjects.

2. One of the main figures for the first part of the paper (Fig. 3) shows the fit for one dataset, while the second dataset is pushed to Supplementary material. It would be clearer to provide a combined plot for both datasets or alternatively explain the rationale for choosing one or the other as a main figure.

3. The table (Table 1) depicting the model comparison seems a bit simple despite supporting one of the main claims of the paper (the need for both stochastic and deterministic retrieval). Is the full model better across the board for all subjects and sessions? Do differences in model fitting depend on the load? Expanding on the model comparison would make a more convincing point about the need for the full model.

4. Despite the qualitatively good fitting of the model to the data, there is a systematic drift where the model slope is steeper than the empirical data at smaller set sizes and flatter at larger set sizes. The authors should provide some logic that helps explain this drift as it is regularly present in all the datasets used.

5. While Supplementary Figure 2 provides some details about the behavioral results, it would be beneficial to expand this information to all datasets. Additionally, including other temporal metrics such as breaks between blocks, trial durations, and response times could help interpret some of the results.

Extra comments

1. Figure 1 schematic could be improved for further clarity as the concepts between and within search are crucial for the rest of the work. The difference between the two is somewhat unclear.

2. Figure 6 is small and hard to read. Some of the terminology (Schematics and Baseline) should be consistently capitalized, and methods should clarify what these refer to.

3. In line 160, the correct reference should be Supp. Fig. 1

**********

**Have the authors made all data and (if applicable) computational code underlying the findings in their manuscript fully available?**

The PLOS Data policy requires authors to make all data and code underlying the findings described in their manuscript fully available without restriction, with rare exception (please refer to the Data Availability Statement in the manuscript PDF file). The data and code should be provided as part of the manuscript or its supporting information, or deposited to a public repository. For example, in addition to summary statistics, the data points behind means, medians and variance measures should be available. If there are restrictions on publicly sharing data or code —e.g. participant privacy or use of data from a third party—those must be specified.

Reviewer #1: Yes

Reviewer #2: **No:** Data is available under request

Reviewer #3: **No:** The code used for the modelling cannot be found (at least not that I could)

**********

PLOS authors have the option to publish the peer review history of their article (what does this mean?). If published, this will include your full peer review and any attached files.

If you choose “no”, your identity will remain anonymous but your review may still be made public.

**Do you want your identity to be public for this peer review?**  For information about this choice, including consent withdrawal, please see our Privacy Policy.

Reviewer #1: No

Reviewer #2: **Yes:** Genis Prat-Ortega

Reviewer #3: **Yes:** Tiffany Ona-Jodar

**Figure resubmission:**

While revising your submission, we strongly recommend that you use PLOS’s NAAS tool (https://ngplosjournals.pagemajik.ai/artanalysis) to test your figure files. NAAS can convert your figure files to the TIFF file type and meet basic requirements (such as print size, resolution), or provide you with a report on issues that do not meet our requirements and that NAAS cannot fix.

**Reproducibility:**

To enhance the reproducibility of your results, we recommend that authors of applicable studies deposit laboratory protocols in protocols.io, where a protocol can be assigned its own identifier (DOI) such that it can be cited independently in the future. Additionally, PLOS ONE offers an option to publish peer-reviewed clinical study protocols. Read more information on sharing protocols at https://plos.org/protocols?utm_medium=editorial-email&utm_source=authorletters&utm_campaign=protocols

# Author comment: Author response to Decision Letter 1

**DOI:** 10.1371/journal.pcbi.1014535.r002

5 May 2026

Attachment Submitted filename: Response to Reviewers.docx

# Peer review: Decision Letter 1

**DOI:** 10.1371/journal.pcbi.1014535.r003

**Authors:** Tianming Yang, Jorge Mejias

5 Jul 2026

Dear Aponte,

We are pleased to inform you that your manuscript 'Beyond memory capacity: A probabilistic, dual store model of visuospatial working memory' has been provisionally accepted for publication in PLOS Computational Biology.

Before your manuscript can be formally accepted you will need to complete some formatting changes, which you will receive in a follow up email. A member of our team will be in touch with a set of requests.

Please note that your manuscript will not be scheduled for publication until you have made the required changes, so a swift response is appreciated.

IMPORTANT: The editorial review process is now complete. PLOS will only permit corrections to spelling, formatting or significant scientific errors from this point onwards. Requests for major changes, or any which affect the scientific understanding of your work, will cause delays to the publication date of your manuscript.

Should you, your institution's press office or the journal office choose to press release your paper, you will automatically be opted out of early publication. We ask that you notify us now if you or your institution is planning to press release the article. All press must be co-ordinated with PLOS.

Thank you again for supporting Open Access publishing; we are looking forward to publishing your work in PLOS Computational Biology.

Best regards,

Jorge F. Mejias, Ph.D.

Academic Editor

PLOS Computational Biology

Tianming Yang

Section Editor

PLOS Computational Biology

***********************************************************

Reviewer's Responses to Questions

**Comments to the Authors:**

**Please note here if the review is uploaded as an attachment.**

Reviewer #2: The authors have addressed all my comments. I only have a couple of clarification comments. Congratulations for the nice study.

In the multistrore model (line 190), Is the probability of a BSE a linear function of the number of items shared (line 190) or the load ( as suggested in the legend of figure 2D)?

In the multistore model, is L equivalent to B?

There is a similar issue in line 212

If I understand correctly, it seems that in the multistore model the items are stored in the deterministic/ “save” memory and only when M_B is full, they are stored in the M_stoch. A prediction of that model will be that the items stored early will be always retrieved while items stored later will not, which seems counterintuitive. Do you find something like that in the data? This effect might be acknowledged as a limitation in the discussion when the authors talk about the “recency” effect but it is not clear. A clarification could improve the discussion.

Reviewer #3: I thank the authors for their thoughtful and comprehensive responses to my comments. Although some of the points raised did not lead to major changes in the overall results or methodology, I appreciate the additional analyses and discussion addressing potential limitations and generally increasing the transparency of the results. These additions provide important context for interpreting the findings and strengthen the manuscript.

Overall, I am pleased with the revisions and feel that my concerns have been adequately addressed. I have no major remaining concerns and believe the manuscript is suitable for publication.

**********

**Have the authors made all data and (if applicable) computational code underlying the findings in their manuscript fully available?**

The PLOS Data policy requires authors to make all data and code underlying the findings described in their manuscript fully available without restriction, with rare exception (please refer to the Data Availability Statement in the manuscript PDF file). The data and code should be provided as part of the manuscript or its supporting information, or deposited to a public repository. For example, in addition to summary statistics, the data points behind means, medians and variance measures should be available. If there are restrictions on publicly sharing data or code —e.g. participant privacy or use of data from a third party—those must be specified.

Reviewer #2: Yes

Reviewer #3: None

**********

PLOS authors have the option to publish the peer review history of their article (what does this mean?). If published, this will include your full peer review and any attached files.

If you choose “no”, your identity will remain anonymous but your review may still be made public.

**Do you want your identity to be public for this peer review?**  For information about this choice, including consent withdrawal, please see our Privacy Policy.

Reviewer #2: No

Reviewer #3: **Yes:** Tiffany Ona-Jodar

# Editor report: Acceptance letter

**DOI:** 10.1371/journal.pcbi.1014535.r004

**Authors:** Tianming Yang, Jorge Mejias

PCOMPBIOL-D-25-02350R1

Beyond memory capacity: A probabilistic, dual store model of visuospatial working memory

Dear Dr Aponte,

I am pleased to inform you that your manuscript has been formally accepted for publication in PLOS Computational Biology. Your manuscript is now with our production department and you will be notified of the publication date in due course.

The corresponding author will soon be receiving a typeset proof for review, to ensure errors have not been introduced during production. Please review the PDF proof of your manuscript carefully, as this is the last chance to correct any errors. Please note that major changes, or those which affect the scientific understanding of the work, will likely cause delays to the publication date of your manuscript.

Soon after your final files are uploaded, unless you have opted out, the early version of your manuscript will be published online. The date of the early version will be your article's publication date. The final article will be published to the same URL, and all versions of the paper will be accessible to readers.

For Research, Software, and Methods articles, you will receive an invoice from PLOS for your publication fee after your manuscript has reached the completed accept phase. If you receive an email requesting payment before acceptance or for any other service, this may be a phishing scheme. Learn how to identify phishing emails and protect your accounts at https://explore.plos.org/phishing.

Thank you again for supporting PLOS Computational Biology and open-access publishing. We are looking forward to publishing your work!

With kind regards,

Anitha Samidurai

PLOS Computational Biology | Carlyle House, Carlyle Road, Cambridge CB4 3DN | United Kingdom ploscompbiol@plos.org | Phone +44 (0) 1223-442824 | ploscompbiol.org | @PLOSCompBiol
